# Towards a Resilience-Oriented Framework for Fault Diagnosis Under Varying Operating Conditions

**DOI:** 10.3390/s26165239

**Published:** 2026-08-19

**Authors:** Nada Baddou, Afaf Dadda, Bouchra Rzine

**Affiliations:** 1 Industrial Techniques Laboratory (LTI), Faculty of Science and Technology, Sidi Mohamed Ben Abdellah University, Fes P.O. Box 2202, Morocco; bouchra.rzine@usmba.ac.ma; 2 Mathematical and Computer Modeling Laboratory, National Higher School of Arts and Crafts (ENSAM), University Moulay Ismail, Meknes 50500, Morocco; a.dadda@ensam.umi.ac.ma

**Keywords:** predictive maintenance, Industry 5.0, fault diagnosis, resilience, confidence-aware, multi-sensor data fusion, deep learning, variable operating conditions, deployability assessment

## Abstract

Achieving high fault-classification accuracy alone does not guarantee reliable autonomous operation under varying operating conditions, raising the need to assess prediction reliability and deployment readiness. This work proposes a resilience-oriented framework for fault diagnosis under varying operating conditions, characterizing diagnostic behavior under operating-condition shifts and providing complementary information on confidence, deployability, and supervision requirements. The framework fuses multi-sensor vibration and motor current signals within a Multi-Stage architecture combining a data-driven branch (DD-MSCNN) and a physics-aware branch (PA-MSCNN) integrating order-tracking descriptors, augmented by a confidence-aware deployability assessment layer. Evaluated on the Paderborn KAT dataset across six bidirectional shifts involving speed, torque, and radial force, the results reveal that operating-condition shifts are not equivalent and that their impact is strongly direction-dependent. Physical knowledge does not systematically guarantee superior performance, highlighting the complementary roles of the two representations. To formalize these observations, the Physics Contribution Index (PCI), the Shift Directionality Index (SDI), and a four-level deployability classification are introduced, providing quantitative insights into prediction reliability and autonomous operation readiness in dynamic industrial environments.

## 1. Introduction

Predictive maintenance has established itself as one of the most tangible expressions of Industry 4.0, a discipline where data-driven methods and artificial intelligence converge to anticipate equipment failures before they occur, reducing unplanned downtime and maintenance costs [1]. As industrial systems grow more connected and instrumented, the volume of operational data available for condition monitoring has expanded considerably, enabling increasingly sophisticated diagnostic models. Yet the transition toward Industry 5.0 introduces a broader set of requirements that go beyond prediction accuracy. Where Industry 4.0 prioritized automation and data efficiency, Industry 5.0 reframes the relationship between humans and intelligent systems around three interconnected pillars: human-centricity, sustainability, and resilience [2,3].

Among these three pillars, resilience represents one of the most critical challenges for industrial fault diagnosis. These pillars are not independent in dynamic industrial environments; they reinforce one another. A resilient diagnostic system must not only perform well under nominal conditions but also adapt to changing operating regimes, communicate its uncertainty honestly, and collaborate with the human operator when its confidence is insufficient.

At the conceptual level, resilience, in the context of fault diagnosis, can initially be understood as the capacity of a model to maintain reliable performance when the conditions under which it operates deviate from those under which it was trained. Industrial equipment does not run at a fixed speed, load, or configuration; motors accelerate and decelerate, production demands fluctuate, and maintenance interventions alter the mechanical state of rotating components. A model trained to recognize fault signatures at 900 rpm might fail to recognize the same fault at 1500 rpm, not because the fault has changed, but because its physical manifestation has shifted [4]; crucially, different types of operating-condition change do not affect fault signatures in the same way, and understanding this distinction is not merely academic: it determines where a diagnostic system will fail in deployment, and what kind of intervention can correct it.

The methods developed for bearing fault detection have evolved considerably over the past decade, progressing from classical machine learning approaches to deep learning architectures with increasingly expressive representations [1]. Convolutional neural networks, long short-term memory networks, attention mechanisms, and transformer-based models have each extended the diagnostic frontier, pushing accuracy metrics higher on benchmark datasets [5]. More recently, deep reinforcement learning has introduced a new learning paradigm: agents that learn decision policies through interaction with an environment, optimizing cumulative reward over time [6]. This paradigm suits the sequential, cost-asymmetric nature of fault diagnosis. Digital twins, virtual replicas enabling simulation-based exploration of operating configurations before deployment, extend this potential further [7].

Beyond learning architectures, the quality and complementarity of the sensed information also play a central role. The sensors that feed these diagnostic systems have undergone parallel advances. Vibration accelerometers have long been the dominant modality for bearing fault detection, offering direct measurement of the impulsive mechanical events that characterize rolling element defects [8]. However, recent work has begun to explore the complementary value of motor phase current signals. Bearing defects generate periodic torque fluctuations that modulate the phase current at characteristic fault frequencies, producing sideband components in the current spectrum that are largely invisible to global amplitude metrics but detectable through learned multi-scale representations [9]. Research has demonstrated that multi-sensor fusion achieves better diagnostic results than single-sensor approaches, as the complementary nature of vibration and current signals provides richer fault representations [10]. Critically, current signal amplitude, directly proportional to power consumption, opens a pathway toward energy monitoring. Recent industrial audits have identified fleet-level energy losses exceeding 17% in motor systems operating with undetected degradation [11], connecting early fault detection directly to the sustainability pillar of Industry 5.0.

Beyond architectural performance, reliable industrial deployment requires diagnostic models not only to predict accurately but also to quantify the confidence of their predictions, so that unreliable outputs can be identified before they lead to erroneous maintenance decisions [12]. Recent works have addressed this need through Bayesian deep learning for uncertainty quantification and confidence calibration [13], and through uncertainty-aware deep ensembles combined with out-of-distribution detection [14]. However, most of these approaches are designed and evaluated under matched operating conditions, leaving their behavior under cross-condition distribution shifts, where confidence calibration becomes most critical, largely unexplored.

Despite these advances, a fundamental gap persists in the literature. The vast majority of published diagnostic models are trained and evaluated under static, matched operating conditions, with the same speed, load, and configuration in both training and test sets [15]. This evaluation protocol does not reflect the reality of industrial deployment, where the conditions encountered after model training rarely match those under which the model was built. The consequence manifests in two directions: false positives, where a change in operating condition is misinterpreted as a fault, eroding operator trust; and false negatives, where a genuine fault is masked by an unfamiliar signal context, allowing damage to progress undetected. A model that achieves 96% accuracy on a benchmark may degrade significantly when deployed under a different rotational speed, not because it has been poorly designed, but because it was never asked to generalize [16]. Consequently, existing studies mainly answer whether a model can classify faults accurately, but provide little guidance on whether it can be safely deployed under unseen operating conditions.

To address this gap, this study proposes a two-stage confidence-aware fault diagnosis framework for rotating machinery. While the framework is general by design, it is instantiated and validated on rolling-element bearings, which constitute one of the most critical and failure-prone components in rotating machinery. The framework is evaluated under three physically distinct types of operating-condition shift, rotational speed, load torque, and radial force variation, using the publicly available Paderborn KAT benchmark [17]. The framework fuses vibration and motor phase current signals through a multimodal deep learning architecture, separating fault detection from fault classification in a deliberate two-stage design. A confidence estimator derived from the classifiers’ softmax outputs characterizes the reliability of each prediction under cross-condition deployment, following the selective prediction (reject-option) paradigm [18]. Rather than accepting every prediction indiscriminately, the framework quantifies, through an Escalation Candidate Rate, the proportion of decisions that cannot be considered sufficiently reliable for fully autonomous acceptance and that would therefore warrant additional supervision. The results show that this behavior is physically governed: frequency-altering perturbations generate uncertain predictions that are correctly flagged as low-confidence, whereas amplitude-altering perturbations generate confident errors that signal a physical boundary requiring a different mitigation strategy, an honest acknowledgment that, we argue, is a prerequisite for trustworthy deployment in Industry 5.0 environments.

Despite a decade of architectural progress, the gap between laboratory accuracy and industrial reliability remains largely unaddressed. Many diagnostic models are still evaluated under matched or distribution-mixed conditions, whereas practical deployment often requires operation in regimes that were not available during training. Moreover, many cross-condition approaches rely on target-domain information, such as unlabeled recordings, pseudo-labels, or distributional statistics, to reduce the source-target discrepancy. In real industrial settings, however, such information is not always available before deployment. This work therefore approaches bearing fault diagnosis as a target-free cross-condition problem: a model is trained on a single source operating condition, without access to target-condition data, measurements, labels, or distributional information, in any form, and evaluated on previously unseen operating conditions. Within this setting, the central question is not only whether a fault can still be recognized, but also whether the diagnosis remains reliable enough for autonomous deployment, how the direction of the operating-condition shift affects performance, and when additional supervision or adaptation may be required. These questions structure the present study and are formalized as follows:

RQ1: How do data-driven and physics-aware representations behave under target-free operating-condition shifts?

RQ2: Under which operating-condition shifts do physics-aware descriptors improve diagnostic robustness?

RQ3: Are operating-condition shifts intrinsically directional in their impact on diagnostic performance?

RQ4: How can confidence information and disagreement between complementary representations provide additional insight into the reliability of cross-condition diagnostic decisions?

To the best of our knowledge, few works formalize target-free cross-condition deployability under bidirectional operating-condition shifts using explicit indicators that jointly combine robustness, physical contribution, shift directionality, and supervision requirements. The main contributions of this work are summarized as follows:

- A multi-stage cross-condition fault diagnosis framework integrating an early-warning stage, a parallel fault classification stage based on complementary data-driven and physics-aware representations, and a resilience and deployability assessment stage to support operation under unseen operating conditions.
- A Physics Contribution Index (PCI) that quantifies the contribution of physical knowledge to diagnostic robustness across operating-condition shifts and fault categories.
- A Shift Directionality Index (SDI) that characterizes the asymmetry between forward and reverse operating-condition transitions and highlights the directional nature of distribution shifts.
- A confidence-aware deployability assessment framework introducing the Escalation Candidate Rate (ECR) and deployability classes, providing quantitative guidelines regarding autonomous deployment, supervision requirements, and adaptation needs.

The remainder of this paper is organized as follows: Section 2 reviews the existing literature on deep-learning-based bearing fault diagnosis, diagnosis under variable operating conditions, and confidence-aware and resilient diagnostic frameworks. Section 3 presents the proposed framework, including the Multi-Stage diagnostic strategy, the dual data-driven and physics-aware representations, and the confidence-aware and deployability assessment layer. Section 4 describes the experimental setup, dataset, and evaluation protocol. Section 5 reports and discusses the experimental results obtained under identical and varying operating conditions, with particular emphasis on the six bidirectional operating-condition shifts. Finally, Section 6 concludes the paper and outlines directions for future research.

## 2. Related Work

### 2.1. Deep Learning Approaches Used in Bearing Fault Diagnosis

Rolling bearings are critical components in rotating machinery, and their failure represents one of the most frequent and costly sources of industrial downtime. Early and reliable fault detection has therefore become a central objective in predictive maintenance research, driving the development of increasingly sophisticated diagnostic methods over the past decade [19].

**Figure 1:**
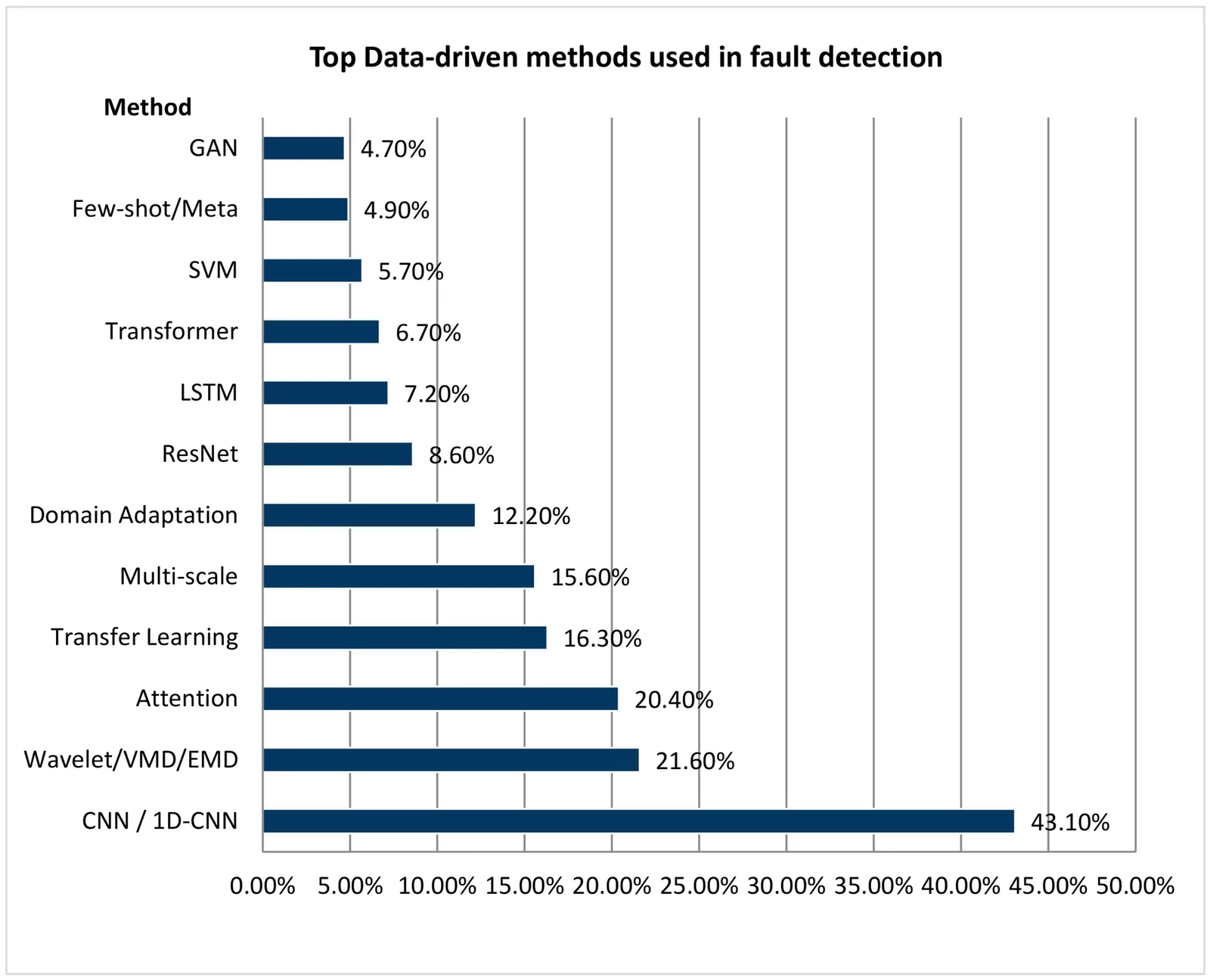
Top data-driven methods used for fault diagnosis.

To provide a quantitative overview of the existing approaches in this field, a systematic search was conducted on Scopus in May 2026 combining keywords related to bearing fault diagnosis and data-driven methods, returning 2529 journal articles published between 2020 and 2026. Figure 1 summarizes the distribution of the main methods identified in this corpus.

As shown in Figure 1, Convolutional neural networks (CNN)-based architectures appear in 43.1% of the reviewed works, making them the dominant feature extraction paradigm. Signal processing preprocessing methods such as wavelet transforms [20] and Variational Mode Decomposition (VMD) [21] appear in 21.6% of articles, attention mechanisms [22] in 20.4%, transfer learning [23] in 16.3%, and transformer-based models [24] in 6.7%, with the latter showing rapid adoption after 2023. Classical machine learning approaches such as SVM [25] remain marginal (5.7%), confirming a near-complete but not total transition toward end-to-end deep learning [26]. The predominance of CNN-based architectures demonstrates that architectural innovation remains the dominant research direction. However, comparatively fewer studies address deployment reliability under operating-condition shifts, which motivates the present work.

CNN emerged as the core feature extraction architecture in this transition, demonstrating strong capabilities for automatically learning hierarchical fault representations from raw vibration signals without manual feature engineering [27,28]. To capture the temporal structure of fault signatures, which manifest as periodic impulse patterns whose dynamics evolve over successive signal windows, CNN architectures were progressively combined with recurrent modules. The Long Short-Term Memory network (LSTM), a recurrent architecture of the neural network family that selectively retains and forgets information across time steps through gating mechanisms, specifically designed to learn long-range temporal dependencies, proved particularly effective when combined with multi-scale convolutional layers [29,30]. Bidirectional variants, which process sequences in both forward and backward directions, improving sensitivity to asymmetric fault patterns, further extended this design pattern [31]. The introduction of attention mechanisms, modules that weight input features according to their diagnostic relevance by computing similarity scores across signal segments, added a further dimension, enabling models to focus selectively on fault-indicative portions of the signal [32].

More recently, transformer-based architectures have extended the diagnostic frontier by replacing recurrent modules with self-attention mechanisms that capture long-range temporal dependencies without the vanishing gradient limitations of LSTM networks [33,34]. Although transformers were absent from the bearing fault diagnosis literature before 2021, they appear in 12.3% of 2025 publications, signaling a rapid architectural shift toward global context modeling.

Despite this architectural diversity, a fundamental methodological concern persists across the literature. Ref. [35] demonstrates that window-level splitting between training and test sets introduces systematic data leakage, allowing signals from the same physical recording to contaminate both sets, thereby inflating reported performance metrics. Furthermore, the vast majority of published evaluations are conducted under matched operating conditions, with the same speed, load, and configuration in both training and test sets, a protocol that does not reflect the dynamic variability of real industrial environments. A model that achieves 96% accuracy under matched conditions may degrade significantly when the operating regime changes between training and deployment [36]. This gap between laboratory evaluation and industrial applicability constitutes the primary motivation for the approach developed in the present work.

### 2.2. Fault Diagnosis Under Variable Operating Conditions

A prerequisite for any meaningful discussion of model generalization is a precise characterization of what “variable operating conditions” means in practice, a distinction that is frequently overlooked in the published literature and that has direct consequences for the interpretation of reported results. Three fundamentally different experimental protocols coexist under this label, each reflecting a different assumption about what the model is expected to learn.

The first and most prevalent protocol, which we term distribution mixing, pools data from multiple operating conditions into a single training set and evaluates the model on a held-out subset drawn from the same mixed distribution. Under this protocol, the model implicitly encounters the full range of condition variability during training, and its generalization is tested within a distribution it has already partially seen. Representative works in this category train and evaluate their models on data drawn from the same mixed pool of operating conditions, regardless of the underlying architecture employed, whether convolutional networks operating on raw signals, recurrent networks, or signal-to-image transformation approaches. The reported accuracy reflects performance within this mixed distribution rather than under truly unseen conditions [8,15]. While effective under this protocol, such approaches do not evaluate the model’s behavior when the operating regime changes after training, the scenario that most closely reflects industrial deployment. The second protocol, cross-condition transfer, trains the model on one or more source conditions and evaluates it on a distinct target condition, with some form of target data made available during training or adaptation, whether labeled samples, unlabeled recordings, or distributional statistics. A spatiotemporal feature fusion domain-adaptive network illustrates this approach, using VMD and FFT [37] to construct joint time-frequency representations and aligning source and target distributions through joint maximum mean discrepancy to address feature inconsistency caused by changing conditions [38]. The third protocol, the most demanding and the most industrially realistic, trains the model on a single source condition and evaluates it on a target condition entirely absent from training in any form. The distinction between these three protocols matters critically: Ref. [35] demonstrates that window-level splitting between training and test sets introduces systematic data leakage, allowing signals from the same physical recording to appear in both sets, thereby inflating reported performance metrics in ways that do not reflect real deployment performance [36].

These three protocols are not equivalent in industrial relevance. Distribution mixing is the least informative: a model trained on a mixed pool does not generalize; it interpolates within a distribution it has already seen, associating fault labels with condition-specific artifacts rather than with intrinsic bearing fault signatures. We term this phenomenon distributed memorization. True generalization can only be measured when the model is evaluated on a condition absent from training in any form: no labeled samples, no unlabeled recordings, no distributional statistics. This target-free protocol is the one adopted in the present work.

A complementary line of work addresses variable-condition robustness through statistically grounded health-indicator design rather than deep representation learning: a slice-oriented signal probability distribution measure has been proposed for wind turbine generator bearing condition monitoring, explicitly compensating for discrepancies induced by variable operating speed [39]. This illustrates that robustness to variable operating conditions can also be pursued through classical probabilistic signal characterization, complementary to the deep-learning-based cross-condition strategies discussed above.

Within the cross-condition transfer paradigm, methods have progressively reduced the dependency on target-domain supervision. MMD-based alignment minimizes distributional discrepancy at the feature level [40,41], joint distribution adaptation extends this to conditional distributions [42], adversarial domain adaptation produces domain-invariant embeddings, and contrastive learning with pseudo-labeling enables fully unsupervised adaptation under time-varying conditions [43]. Source-free domain adaptation further reduces deployment constraints by adapting without retaining source data [44], and has recently been validated on the Paderborn benchmark through dual-stream hybrid-domain adaptation [45]. Physics-aware approaches have also emerged, integrating BPFI and BPFO descriptors into CNN architectures to improve interpretability under variable conditions, yet these still require target-condition data and do not address uncertainty quantification or human–machine collaboration.

Table 1 summarizes the key methodological characteristics of the reviewed variable-condition approaches. As shown, all reviewed approaches, regardless of their methodological sophistication, require access to target-condition data in some form during training or adaptation. The present work is the only evaluated approach that operates without any target-condition data, trained on a single source condition and evaluated on three physically distinct target conditions that are entirely absent from training.

Table 1 therefore highlights that the originality of the present work lies not in proposing another transfer strategy, but in characterizing deployment behavior under target-free operating-condition shifts.

### 2.3. Confidence-Aware and Resilient Fault Diagnosis Frameworks

A growing body of work argues that diagnostic accuracy alone is an insufficient criterion for safety-critical industrial deployment, and that a model must also be able to express when its predictions can be trusted. Deep-learning diagnosis models are often not truly trusted by users because of the lack of interpretability of the “black box”, which limits their deployment in safety-critical applications; a trusted system therefore requires both accurate diagnosis in most cases and a human in the decision-making loop to handle the abnormal situations in which the model fails [12]. This formulation of high autonomy in the nominal regime and human escalation in the uncertain regime underpins most recent confidence-aware designs and motivates the confidence-driven escalation mechanism developed in the present work, in which uncertain predictions are flagged rather than accepted autonomously. The same principle has recently been generalised beyond fault diagnosis: a review in Discover Artificial Intelligence contends that transparency and explainability can foster trust only when combined with mechanisms for uncertainty communication and trust calibration, proposing the TEUT [47].

Several strategies have been proposed to equip diagnostic models with a calibrated sense of confidence, most of them probabilistic. A representative example integrates Bayesian deep-learning techniques with model-calibration strategies, using a Bayesian convolutional backbone in which α-divergence decomposes and quantifies epistemic and aleatoric uncertainty, achieving out-of-distribution sample detection through the epistemic component while calibration constraints improve the confidence of in-distribution diagnosis [48]. Closely related, a trustworthy Bayesian framework couples uncertainty quantification with confidence calibration for machinery fault diagnosis, noting that traditional intelligent methods focus only on the accuracy of in-distribution samples while neglecting the trustworthiness of the results [13]. Because full Bayesian inference is computationally demanding, evidential alternatives have emerged that incorporate a stacked gated recurrent unit into an evidential neural network to quantify prediction uncertainty and estimate diagnostic trustworthiness, validated on rolling-bearing fault diagnosis under variable noise conditions [49].

A complementary line of research converts this quantified uncertainty into an explicit decision rule rather than leaving it as a diagnostic by-product. One such approach establishes an ensemble of deep neural networks whose uncertainty-aware analysis detects out-of-distribution samples and issues warnings for potentially untrustworthy diagnoses, with a selection criterion for the uncertainty threshold so that trustworthy decisions combine both the ensemble prediction and its uncertainty [14]. This threshold-gated escalation is conceptually close to the deployability classes and verification-rate logic developed later in this paper.

Beyond data-driven fault diagnosis, recent research has explored stochastic computational frameworks to address uncertainty in engineering systems, particularly for probabilistic characterization of material and structural variability [50]. Although these approaches address different objectives from the present work, they further illustrate the increasing interest in uncertainty-aware methodologies.

A second, largely parallel research stream targets resilience, the ability of a diagnostic model to maintain stable performance when the operating conditions of the machine change. This is a central obstacle to industrial deployment, since the mismatch between training and operating conditions is widely framed as a distribution-shift problem in which future operating regimes diverge from those observed during training. Architectural robustness offers one response: multi-scale convolutional designs have been shown to enhance resilience to signal noise and to variations in operating conditions through a multi-scale architecture combined with depth-wise feature concatenation [51], a design philosophy shared by the MSCNN backbone adopted here. At the evaluation level, recent surveys call for assessment that goes beyond diagnostic accuracy to systematically consider robustness on out-of-distribution data, computational efficiency, and integration within real industrial systems, alongside cross-condition robustness and real-time capability [52].

Despite this progress, the two streams have largely developed in isolation: confidence-aware methods quantify when to trust a prediction but are typically validated under fixed or distribution-mixed conditions, whereas resilience-oriented methods address how performance degrades across conditions but rarely translate that degradation into an operational confidence or escalation criterion. Moreover, robustness is almost always reported as an aggregate accuracy figure, which conceals both the per-fault structure of the errors and the direction of the operating-condition change. The framework proposed in this paper sits deliberately at the intersection of these two strands: it couples a resilience analysis across bidirectional operating-condition shifts with a confidence-driven, deployability-oriented decision layer, so that cross-condition degradation is expressed directly as a quantified, deployment-oriented supervision requirement rather than as a single accuracy score. This gap motivates the unified confidence-aware and deployability-oriented framework proposed in this work.

## 3. Proposed Framework

### 3.1. Overall Framework Architecture

**Figure 2:**
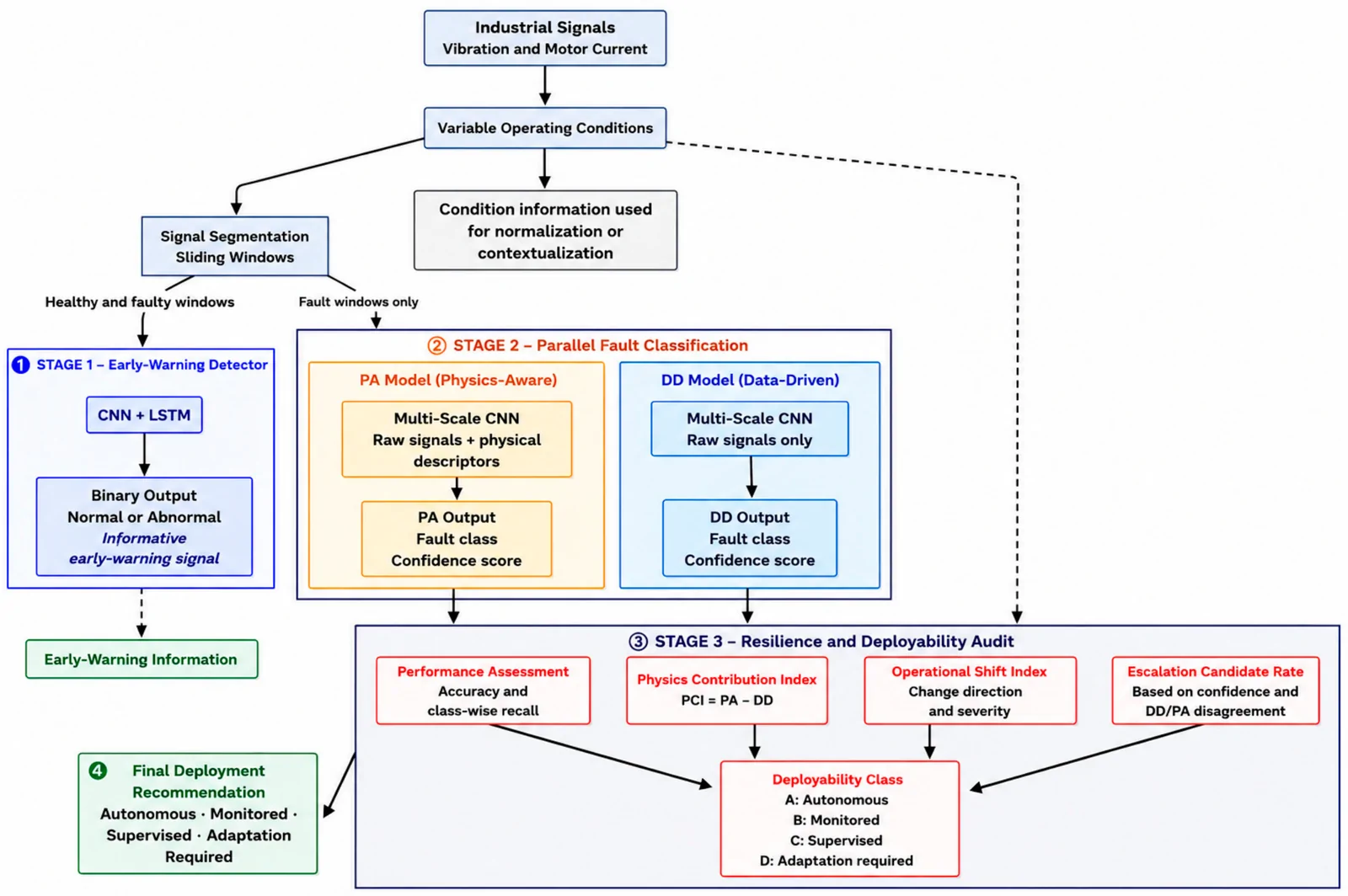
Overall architecture of the proposed confidence-aware and deployability-oriented fault diagnosis framework.

The overall architecture of the proposed framework is illustrated in Figure 2. The framework is organized into five main modules: multi-sensor signal acquisition, Multi-Stage diagnosis, dual representation learning, confidence-aware decision making, and deployability assessment.

The first diagnostic stage performs fault detection by distinguishing healthy operating conditions from faulty operating conditions and provides an informative early-warning signal. In parallel, the second diagnostic stage focuses exclusively on detailed fault classification and considers only faulty samples to discriminate between the different fault categories considered in this study.

To support this classification task, two complementary feature extraction branches are employed. The DD-MSCNN branch extracts discriminative representations directly from the multi-sensor signals using a Multi-Scale Convolutional Neural Network (MSCNN). In parallel, the PA-MSCNN branch extracts descriptors derived from bearing kinematics and order-tracking analysis. The outputs of these two branches are independently used to generate diagnostic predictions.

The resulting predictions are subsequently analyzed by a confidence-aware decision layer. This module compares the outputs of the two representations and evaluates their consistency before producing a final diagnostic decision.

Finally, the framework includes a deployability assessment module that evaluates the behavior of the diagnostic system under operating-condition changes. This module generates the indicators used throughout this work to analyze robustness, decision reliability, and deployment readiness.

The individual components of the framework are detailed in the following subsections.

Unlike conventional fault-diagnosis pipelines that terminate at class prediction, the proposed framework extends the diagnostic process with a confidence-aware deployability assessment layer that explicitly characterizes deployment readiness under operating-condition shifts.

Industrial vibration and motor-current signals, acquired under variable operating conditions, are segmented into sliding windows. Stage 1 (Early-Warning Detector) performs binary healthy/faulty screening using a CNN + LSTM classifier, producing an informative early-warning signal. Windows flagged as faulty proceed to Stage 2 (Parallel Fault Classification), where two independent branches operate in parallel: the PA Model (Multi-Scale CNN combined with physical descriptors) and the DD Model (Multi-Scale CNN using raw signals only), each producing a fault-class prediction and a confidence score. The Resilience and Deployability Audit stage combines four indicators computed from these outputs: Performance Assessment (accuracy and class-wise recall), the Physics Contribution Index (PCI = PA − DD), the Operational Shift Index (capturing the direction and severity of the operating-condition change), and the Escalation Candidate Rate (ECR, derived from prediction confidence and DD/PA disagreement). These indicators jointly determine the Deployability Class (A: Autonomous, B: Monitored, C: Supervised, D: Adaptation Required) and the corresponding Final Deployment Recommendation.

### 3.2. Problem Formulation

Fault diagnosis in rotating machinery is particularly challenging when operating conditions vary over time. Changes in speed, torque, and mechanical loading modify the characteristics of vibration and current signals, altering the visibility and separability of fault-related signatures; a diagnostic model developed under a given operating regime may therefore experience a reduction in reliability when exposed to different conditions.

The problem considered in this work therefore extends beyond fault identification itself to maintaining reliable diagnostic performance under operating-condition shifts, which affect fault signatures unevenly rather than uniformly. The objective of the proposed framework is accordingly to support reliable fault diagnosis while explicitly evaluating the reliability of the resulting decisions under this variability.

### 3.3. Data Preparation and Signal Representation

Prior to feature extraction and diagnosis, the acquired multi-sensor signals undergo a preprocessing stage designed to generate standardized and homogeneous inputs. The framework operates on three synchronized sensing modalities, namely the vibration signal and two motor current signals. Each signal is standardized using z-score normalization (zero mean, unit variance), with the mean and standard deviation computed exclusively from the training data and subsequently applied to both training and testing samples in order to avoid information leakage from the target operating condition.

The continuous signals are then segmented using an overlapping sliding-window strategy, which divides each measurement into fixed-length windows, allowing the model to focus on localized fault-related patterns while increasing the number of available training samples and preserving temporal continuity. The specific window length and stride values, together with the resulting number of windows, are reported in the experimental setup (Section 4). After segmentation, the vibration and current windows are synchronized into a unified multi-sensor representation X = [$X_{vib},X_{I_{1}},X_{I_{2}}]$, where vib, I_1_, and I_2_ denote the vibration signal and the two motor-current segments, respectively. This representation constitutes the common input of both the DD-MSCNN and PA-MSCNN branches.

### 3.4. Multi-Stage Diagnostic Strategy

Fault diagnosis is commonly formulated as a multi-class classification problem in which all health states are identified simultaneously. While such an approach is straightforward, it requires the model to perform fault detection and fault identification within a single decision process. In industrial applications, however, these two objectives do not necessarily have the same importance or level of complexity. Detecting the presence of an abnormal condition is often the first priority, whereas determining the exact fault type represents a subsequent diagnostic task.

To address this issue, the proposed framework adopts a Multi-Stage diagnostic strategy composed of two complementary stages. Rather than performing fault detection and fault-type identification within a single decision process, the two tasks are addressed separately in order to account for their different objectives and levels of complexity.

The first stage focuses on fault detection and aims to distinguish normal operating behavior from abnormal behavior. By reducing the problem to a binary decision, this stage provides an informative early-warning signal regarding the presence of an emerging fault. Such information may support maintenance monitoring by indicating the occurrence of abnormal behavior without requiring detailed fault characterization.

The second stage is dedicated to fault-type classification and is evaluated on faulty samples only, allowing the classifier to focus exclusively on the discrimination between different fault categories. This separation reduces the complexity of the decision space and enables the learning process to concentrate on the subtle differences between fault signatures rather than simultaneously handling fault occurrence detection and fault-type identification.

The Multi-Stage organization therefore serves both operational and modeling objectives. From an operational perspective, it combines early-warning information with detailed fault characterization. From a modeling perspective, it decomposes the overall diagnosis problem into two simpler and more specialized tasks, allowing each stage to be optimized according to its specific objective.

#### 3.4.1. Fault Detection (Stage 1)

The first stage of the proposed framework is dedicated to fault detection and aims to determine whether the monitored machine operates under a healthy or a faulty condition. Unlike conventional laboratory settings, the detector is trained and deployed under different operating conditions. Consequently, the model must distinguish degradation-related signatures from variations induced by changes in speed, torque, or radial load. This distinction is particularly important because operating-condition changes may significantly modify vibration and current signals even when the bearing remains in a good state.

To address this challenge, the vibration signal and the two motor-current signals are processed in parallel by three convolutional feature-extraction branches, one per sensing modality. Each branch automatically learns representative patterns from its corresponding signal, and the resulting representations are concatenated into a unified multi-sensor feature vector that jointly describes the machine operating condition at each time instant.

Rather than performing the decision on isolated observations, the framework exploits the temporal evolution of consecutive feature vectors. The fused representations are arranged into short sequences and processed by a Long Short-Term Memory (LSTM) network with 64 hidden units, which captures the temporal consistency of the extracted patterns and enables the detector to distinguish persistent degradation signatures from transient fluctuations. The final LSTM representation is passed through a fully connected layer and a softmax classifier that estimates the probability of the two operating states, healthy or faulty.

#### 3.4.2. Fault Classification (Stage 2)

The second stage is dedicated to detailed fault characterization and aims to discriminate among the different fault categories present in the data. In contrast to the binary decision performed in Stage 1, this stage focuses exclusively on fault-type discrimination and is evaluated on faulty samples only. The specific fault categories considered in this study are defined in Section 4.

To enhance robustness under varying operating conditions, the proposed framework adopts a dual-representation strategy. Two complementary representations are generated from the same multi-sensor observations. The first representation relies exclusively on the measured signals and constitutes the data-driven branch. The second representation integrates additional physics-aware descriptors derived from order tracking analysis and the characteristic fault frequencies of the monitored component, as described in the following subsection.

### 3.5. Dual Representation Module

To improve diagnostic robustness under varying operating conditions, the proposed framework relies on two complementary feature representations. The first representation is entirely data-driven and learns discriminative fault signatures directly from the measured signals. The second representation incorporates prior physical knowledge through order tracking analysis and characteristic bearing frequencies. Both representations are built upon the same MSCNN signal-encoding backbone and the same training strategy. The physics-aware branch additionally processes the order-domain descriptors through a small dedicated sub-network, whose output is fused with the signal features prior to the final classification layers, thereby enriching the learned signal representation with explicit physical descriptors. Consequently, performance differences between the two branches mainly reflect the contribution of the added physical information rather than differences in the underlying signal-processing capacity.

**Figure 3:**
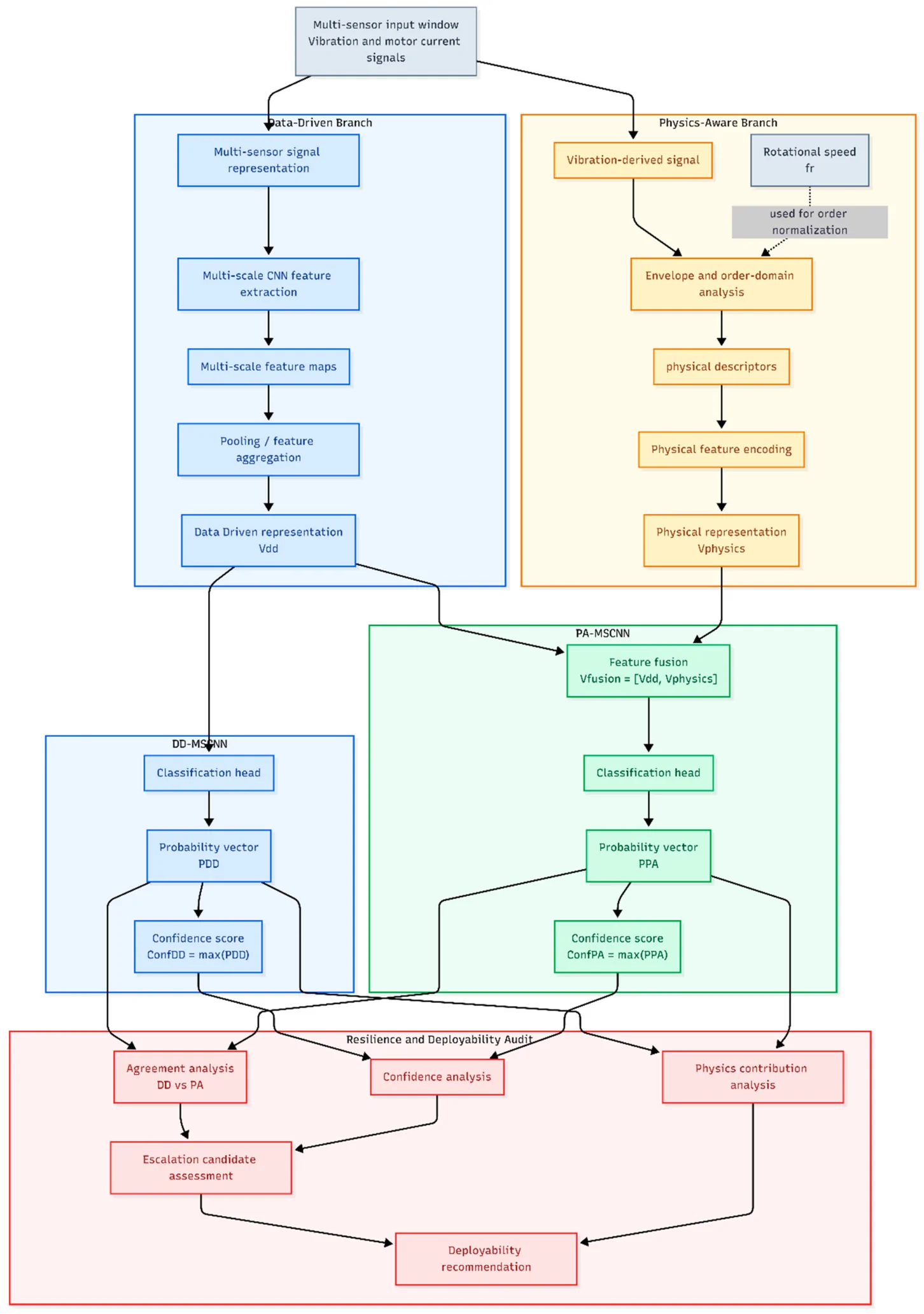
Complementary representation module and resilience-oriented decision layer.

The overall architecture of the two branches, together with their fusion and the subsequent decision layer, is illustrated in Figure 3. Both branches share the same MSCNN signal-encoding backbone, while the physics-aware branch additionally processes order-domain descriptors through a dedicated sub-network before feature fusion. The following subsections detail each branch in turn.

DD-MSCNN (data-driven branch) and PA-MSCNN (physics-aware branch) share the same Multi-Scale Convolutional Neural Network (MSCNN) backbone for encoding the raw vibration and motor-current signals. PA-MSCNN additionally processes order-domain physical descriptors, derived from envelope analysis and characteristic bearing frequencies, through a dedicated sub-network prior to feature fusion. Both branches produce independent fault-class predictions and confidence scores, which are subsequently compared by the confidence-aware decision layer to assess prediction consistency and support the resilience and deployability analysis described in Section 3.6.

#### 3.5.1. Data-Driven Representation

The first branch relies exclusively on the information contained in the measured signals and therefore follows a purely data-driven approach. Its objective is to automatically learn discriminative fault signatures from the multi-sensor observations without introducing any prior physical knowledge.

The multi-sensor input X = [$X_{vib},X_{I_{1}},X_{I_{2}}]$ is processed by a Multi-Scale Convolutional Neural Network (MSCNN). Unlike conventional CNN architectures employing a single receptive field, the MSCNN applies several convolutional branches operating at different kernel sizes in parallel, each capturing fault-related patterns of different durations and frequency contents. The resulting multi-scale feature maps are concatenated into a single data-driven representation, which is then passed through fully connected layers and a softmax classifier to estimate the posterior probability of each fault category. The predicted class is obtained as the category with the highest posterior probability.

This branch therefore performs fault classification solely on the basis of the measured signals, providing a purely data-driven description of the machine condition. The second branch enriches this representation by incorporating additional physical information derived from order-tracking analysis and the characteristic fault frequencies of the monitored component, as described in the following subsection.

#### 3.5.2. Physics-Aware Representation

The physics-aware branch follows a general principle applicable to any rotating component: it enriches the learned representation with descriptors built around the characteristic fault frequencies of the monitored component. Although this principle could in itself be described in a component-agnostic manner, the physical descriptors must ultimately be derived from the specific fault kinematics of the monitored component. Since the present study addresses rolling-element bearings, the branch is necessarily instantiated using bearing-specific vibration characteristics, namely the envelope analysis and order-domain techniques classically employed in vibration-based bearing diagnosis [53]. These tools are not introduced as methodological contributions, but are used to construct physically meaningful descriptors that complement the learned signal representation.

The order-domain representation expresses spectral components relative to the shaft rotational frequency rather than in absolute frequency. For a spectral component at frequency f and a shaft rotational frequency $f_{r}$, the corresponding order is defined as:

(1) $$Order=\frac{f}{f_{r}}$$

where $f_{r}$ denotes the rotational frequency of the shaft. By normalizing frequencies with respect to the rotational speed, order tracking maps the spectral content onto a speed-independent axis: the characteristic fault orders remain at fixed positions regardless of the operating speed. The rotational-speed information is therefore used to perform the normalization, but once the signal is expressed in the order domain, the resulting descriptors become invariant to rotational-speed variations, which facilitates the comparison of signals acquired under different operating conditions.

In the present study, the rotational frequency $f_{r}$ is obtained from the shaft-speed signal acquired synchronously with the vibration and motor-current measurements. For each analysis window, the average rotational speed is used to perform order normalization and to compute the order-domain descriptors.

For rolling-element bearings, the defect-related signatures are associated with the inner-race and outer-race defects, characterized respectively by the Ball Pass Frequency Inner race (BPFI) and the Ball Pass Frequency Outer race (BPFO). Expressed in the order domain introduced above, their characteristic orders are given by:

(2) $$BPFI=\;\frac{n}{2}(1\;+\frac{d}{D}cos\theta )$$

And

(3) $$BPFO=\frac{n}{2}(1-\frac{d}{D}cos\theta )$$

where

*n* is the number of rolling elements;
*d* is the rolling-element diameter;
*D* is the pitch diameter;
*θ* is the contact angle.

These characteristic orders define the locations at which defect-related information is expected to concentrate. Accordingly, an envelope analysis is performed on the vibration signal, and the resulting envelope spectrum is converted into the order domain by normalizing the frequency axis with respect to the shaft rotational frequency. Several physics-aware descriptors are then extracted around the characteristic orders associated with BPFI and BPFO, yielding the following feature vector:

(4) $$P=[e_{BPFI},e_{BPFO},e_{2BPFI},e_{2BPFO},p_{BPFI},\;p_{BPFO},\;r_{1},\;r_{2},K_{env}]$$

where e denotes the normalized energy around each characteristic order and its second harmonic, *p* the corresponding peak amplitudes, *r*_1_ and *r*_2_ the logarithmic energy ratios between the inner- and outer-race orders, and $K_{env}$ the envelope kurtosis. These nine descriptors were selected to jointly capture complementary physical manifestations of bearing defects in the order domain: energy and peak amplitude quantify the strength of the impulsive fault-related components, the order-ratio terms characterize the relative balance between inner- and outer-race contributions, and the envelope kurtosis captures the impulsiveness of the underlying time-domain signal. To reduce the influence of outliers and improve robustness under distribution shifts, these descriptors are normalized using robust statistics.

### 3.6. Confidence-Aware and Deployability Assessment Layer

#### 3.6.1. Agreement and Confidence Analysis

The predictions generated by the data-driven and physics-aware branches are subsequently analyzed by a confidence-aware decision layer. Rather than relying exclusively on a single representation, the proposed framework exploits the complementarity between the two branches to assess the consistency and reliability of the diagnosis under varying operating conditions.

For the data-driven branch, the predicted class is obtained as:

(5) $$y_{DD}\;=\underset{y}{\underbrace{\arg\max\;P_{DD\;}(y|X)}}$$

Similarly, the prediction produced by the physics-aware branch is given by:

(6) $$y_{PA}\;=\underset{y}{\underbrace{\arg\max\;P_{PA}(y|X,P)}}$$

where $P_{DD}(y|X)$ and $P_{PA}(y|X,P)$ denote the class probability distributions estimated by the two branches.

To evaluate the consistency between the two representations, a disagreement indicator is introduced as:

(7) $$D=\left\{\begin{array}{c} \;0,\;y_{DD}=\;y_{PA}\;\\ 1,\;y_{DD}\neq \;y_{PA} \end{array}\right.$$

where (D = 0) indicates that both branches predict the same fault category, whereas (D = 1) corresponds to conflicting predictions. In addition to the predicted labels, the confidence associated with each branch is quantified through the maximum posterior (softmax) probability, which provides a direct and computationally inexpensive estimate of prediction confidence:

(8) $${\;Conf}_{DD}\;=max(P_{DD}\;(y|X))$$

And

(9) $${\;Conf}_{PA}=max(P_{PA\;}(y|X))$$

This confidence score is intentionally lightweight and serves as a practical deployment indicator rather than as a calibrated uncertainty estimator. High confidence values combined with prediction agreement indicate reliable autonomous decisions, whereas low-confidence situations or conflicting predictions reveal increased uncertainty. Such cases are subsequently handled by the resilience-oriented decision mechanisms described in the following subsections.

#### 3.6.2. Escalation Mechanism and Uncertainty Indicators

Although the proposed framework aims to maximize autonomous diagnostic capabilities, uncertain situations may still arise when operating conditions significantly differ from those encountered during training. To prevent unreliable decisions from being accepted automatically, a resilience-oriented escalation mechanism is incorporated into the decision layer.

More specifically, prediction disagreement between the DD-MSCNN and PA-MSCNN branches, together with low-confidence situations, are interpreted as indicators of increased uncertainty. Rather than forcing an automatic decision, such cases are flagged for additional supervision.

The escalation process can therefore be represented by:

(10) $$E=\left\{\begin{array}{c} \;\;\;\;0,\;D=\;0\;and\;conf\;⩾T\;\\ 1,\;D=1\;or\;Conf<T\; \end{array}\right.$$

where (E) denotes the escalation indicator, (D) the disagreement indicator between the DD-MSCNN and PA-MSCNN branches, (Conf) the confidence level associated with the prediction, and (T) a confidence threshold defined in Section 5.4.

Cases associated with (E = 0) are considered sufficiently reliable and are automatically accepted. Conversely, samples corresponding to (E = 1) are regarded as uncertain and may require additional analysis or human supervision. It should be emphasized that samples associated with (E = 1) are not necessarily erroneous predictions. Rather, they correspond to situations for which the framework considers that the available evidence is insufficient to support a fully autonomous decision.

At the scenario level, the proportion of samples associated with (E = 1) is referred to as the Escalation Candidate Rate (ECR). Therefore, the ECR characterizes the fraction of samples considered uncertain and potentially requiring additional verification. A low ECR indicates that the framework is capable of making reliable autonomous decisions, whereas higher ECR values reveal increased uncertainty and a potentially greater need for supervision.

In the present framework, human intervention is not considered as the primary source of performance improvement. Consequently, the ECR should not be interpreted as the actual rate of human intervention. Instead, it reflects the proportion of predictions that are not considered sufficiently reliable to be accepted autonomously.

This mechanism enables the framework to preserve the benefits of autonomous diagnosis while explicitly accounting for uncertainty and limiting the risk of unreliable decisions under previously unseen operating conditions.

Since the confidence values used in this mechanism are obtained directly from the softmax outputs of the two branches, it is worth noting that this choice was made deliberately for its simplicity: it requires no additional training, calibration data, or architectural modification, and it keeps the confidence layer lightweight while ensuring a fair comparison between the data-driven and physics-aware branches, which are assessed under identical confidence criteria.

#### 3.6.3. Physics Contribution Index

Although overall classification accuracy provides a global indication of diagnostic performance, it does not explain whether the incorporation of physical knowledge consistently improves fault diagnosis. Since the proposed framework investigates two complementary representations, additional analysis is required to quantify the actual benefit brought by the physics-aware branch. To this end, a Physics Contribution Index (PCI) is introduced.

The objective is not to redefine diagnostic accuracy, but to isolate the incremental contribution brought by physical descriptors. For a given performance metric, the Physics Contribution Index is defined as:

(11) $$\;PCI={Metric}_{PA\;}\;-{Metric}_{DD}$$

where ${Metric}_{PA}$ and ${Metric}_{DD}$ denote the performances obtained by the physics-aware and data-driven representations, respectively. When accuracy is considered, the corresponding index becomes:

(12) $${PCI}_{Acc}\;={Acc}_{PA}-{Acc}_{DD}$$

Similarly, class-specific contributions can be evaluated by comparing the recall values obtained by the two representations for each class. In the general case, the contribution associated with the $i^{th}$ class is expressed as:

(13) $${PCI}_{{class}_{i}}{=Recall}_{PA}^{{class}_{i}}\;-{Recall}_{DD}^{{class}_{i}}$$

where ${class}_{i}$ denotes the $i^{th}$ class considered in the classification problem.

More generally, for any performance metric M, the contribution associated with the $i^{th}$ class can be written as:

(14) $${{PCI}_{{class}_{i}}^{M}=M}_{PA}^{{class}_{i}}\;-M_{DD}^{{class}_{i}}$$

A positive value of the PCI indicates that the incorporation of physical knowledge improves diagnostic performance, whereas a negative value reveals that the data-driven representation provides superior results. Consequently, the proposed indicator makes it possible to identify the operating conditions and fault categories for which physical descriptors are beneficial, as well as situations in which purely data-driven information remains preferable.

Unlike conventional studies that mainly report absolute classification accuracies, the Physics Contribution Index provides an additional level of interpretation by explicitly quantifying the influence of prior physical knowledge under varying operating conditions.

#### 3.6.4. Shift Directionality Index

Most fault diagnosis studies implicitly assume that the effect of an operating-condition change is independent of its direction. However, the transition from condition A to condition B does not necessarily produce the same diagnostic behavior as the reverse transition from B to A. To explicitly quantify this asymmetry, a Shift Directionality Index (SDI) is introduced.

Although the asymmetry of cross-condition transfer is implicitly acknowledged in the domain-adaptation literature [40], most quantitative domain-discrepancy measures, such as Maximum Mean Discrepancy [41] or the 𝒜-distance, are symmetric by construction and therefore cannot capture whether a forward transfer (A → B) and its reverse (B → A) are equally difficult. The SDI addresses this limitation by quantifying directional asymmetry directly at the level of observed diagnostic performance rather than at the level of feature distributions. Consequently, it enables the assessment of whether opposite operating-condition transitions can genuinely be considered equivalent from a diagnostic perspective.

For a given performance metric, the SDI is defined as:

(15) $$SDI=\frac{\left|{Metric}_{A\to B}-{Metric}_{B\to A}\right|}{\max\left({Metric}_{A\to B},{Metric}_{B\to A}\right)}$$

where Metric A → B and Metric B → A denote the performance achieved under the forward and reverse operating-condition shifts, respectively. Depending on the analysis objective, the metric may correspond to overall accuracy or to class-specific recall values.

A low SDI value indicates that the two directions exhibit similar behavior, suggesting weak directional dependence. Conversely, large SDI values reveal a pronounced asymmetry, meaning that the impact of a shift cannot be inferred from its reverse direction.

Formally, resilience is operationalized in this work through the joint behavior of these three complementary indices. Within the proposed framework, resilience is operationally defined as the joint maintenance, under a given operating-condition shift, of satisfactory diagnostic performance, low directional asymmetry (SDI), and a low escalation rate (ECR). A positive PCI indicates that this robustness is supported by the incorporation of physical descriptors, whereas a negative PCI indicates that the data-driven representation remains more effective under the considered shift, without necessarily implying a lack of resilience. Conversely, degradation along any single indicator identifies a distinct vulnerability: a negative PCI signals a loss of physical relevance when applicable, a high SDI reveals a directional weakness not captured by symmetric evaluation protocols, and a high ECR indicates an unresolved reliability concern requiring human supervision. Accordingly, resilience is interpreted throughout the remainder of this paper through the joint analysis of these complementary indicators rather than through a single aggregated score.

#### 3.6.5. Deployability Assessment and Recommendation

Beyond classification accuracy, practical deployment requires evaluating whether the diagnostic framework can operate autonomously under previously unseen operating conditions. To this end, a deployability assessment module is incorporated into the proposed framework.

Since DD-MSCNN and PA-MSCNN branches may exhibit different behaviors depending on the operating scenario, the best autonomous performance is defined as:

(16) $${Acc}_{best}=\max\left({Acc}_{DD},\;{Acc}_{PA}\right)$$

where ${Acc}_{DD}$ and ${Acc}_{PA}$ denote the accuracies achieved by the data-driven and physics-aware representations, respectively.

In addition, reliable deployment requires maintaining satisfactory performance across all fault categories. Therefore, the weakest class performance is characterized by the minimum class-specific recall:

(17) $${\;Recall}_{worst}=\underset{i}{\underbrace{min({Recall}_{i})}}$$

where ${Recall}_{i}$ represents the recall associated with the $i^{th}$ class.

Based on these indicators, together with the confidence-related information introduced previously, each operating scenario is assigned to one of four deployability categories. In practice, the assessment jointly considers the best autonomous accuracy, the weakest class-specific recall, and the uncertainty level characterized by the Escalation Candidate Rate (ECR).

Class A corresponds to scenarios exhibiting high diagnostic performance together with low uncertainty levels, making autonomous deployment possible. Class B includes scenarios that maintain satisfactory performance but present moderate uncertainty, thus requiring periodic monitoring. Class C represents situations where performance degradation or elevated uncertainty limits fully autonomous operation and closer supervision becomes necessary. Finally, Class D corresponds to severe degradation or strong uncertainty, indicating that additional adaptation or target-condition information is required before deployment.

This categorization enables the framework to move beyond conventional performance reporting by explicitly linking diagnostic performance and confidence indicators to practical deployment capabilities.

Furthermore, the complementary behavior of the data-driven and physics-aware representations is exploited to formulate deployment recommendations. Depending on the values of the Physics Contribution Index, the confidence-related indicators, and the resulting deployability level, the framework may favor the physics-aware representation, the data-driven representation, or indicate that additional target-condition adaptation is required.

Consequently, the proposed decision layer provides not only diagnostic predictions but also quantitative information regarding confidence, resilience, and deployability. Such information is particularly valuable for supporting the practical implementation of intelligent maintenance systems operating under varying industrial environments. The proposed framework therefore answers not only “Which fault is present?” but also “Can this prediction be trusted for autonomous deployment under the considered operating-condition shift?”

## 4. Experimental Setup and Case Studies

### 4.1. Dataset Description

**Figure 4:**
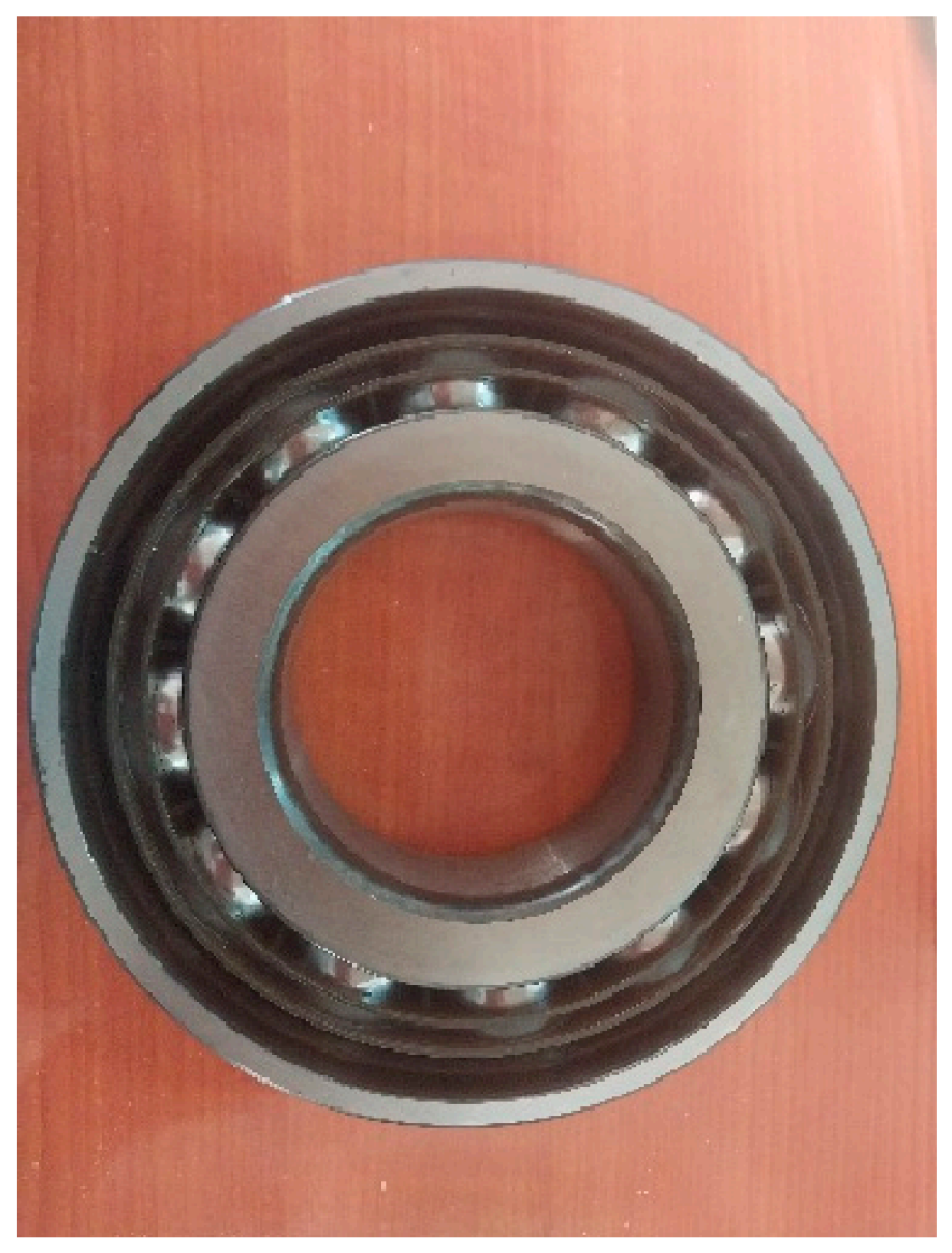
Ball bearing real picture.

Ball bearings are among the most heavily used components in industrial systems. As illustrated in Figure 4, a ball bearing consists of three main elements: the outer ring, the inner ring, and the rolling elements that transmit motion between the two rings. Each of these elements is susceptible to developing defects over time due to the combined effects of mechanical loads, vibrations, and gradual wear. Early detection of these defects is crucial for industrial maintenance, as an undetected bearing failure can result in the unplanned downtime of an entire production line.

To validate the proposed framework under reproducible conditions comparable to the state of the art, we use the public Paderborn KAT dataset, developed by the University of Paderborn and widely used in the predictive maintenance community. This dataset offers several advantages that justify its selection. First, it provides synchronized measurements of multiple sensor modalities, vibration signal and two motor phase current signals, making it particularly well-suited for evaluating multimodal fusion approaches. Second, it covers both artificial and real-world faults induced by accelerated lifetime tests, thus offering a diversity of fault signatures close to real-world industrial conditions. Third, the experiments are conducted under several controlled operating conditions, allowing us to assess the robustness of the models to changes in operating regime.

**Figure 5:**
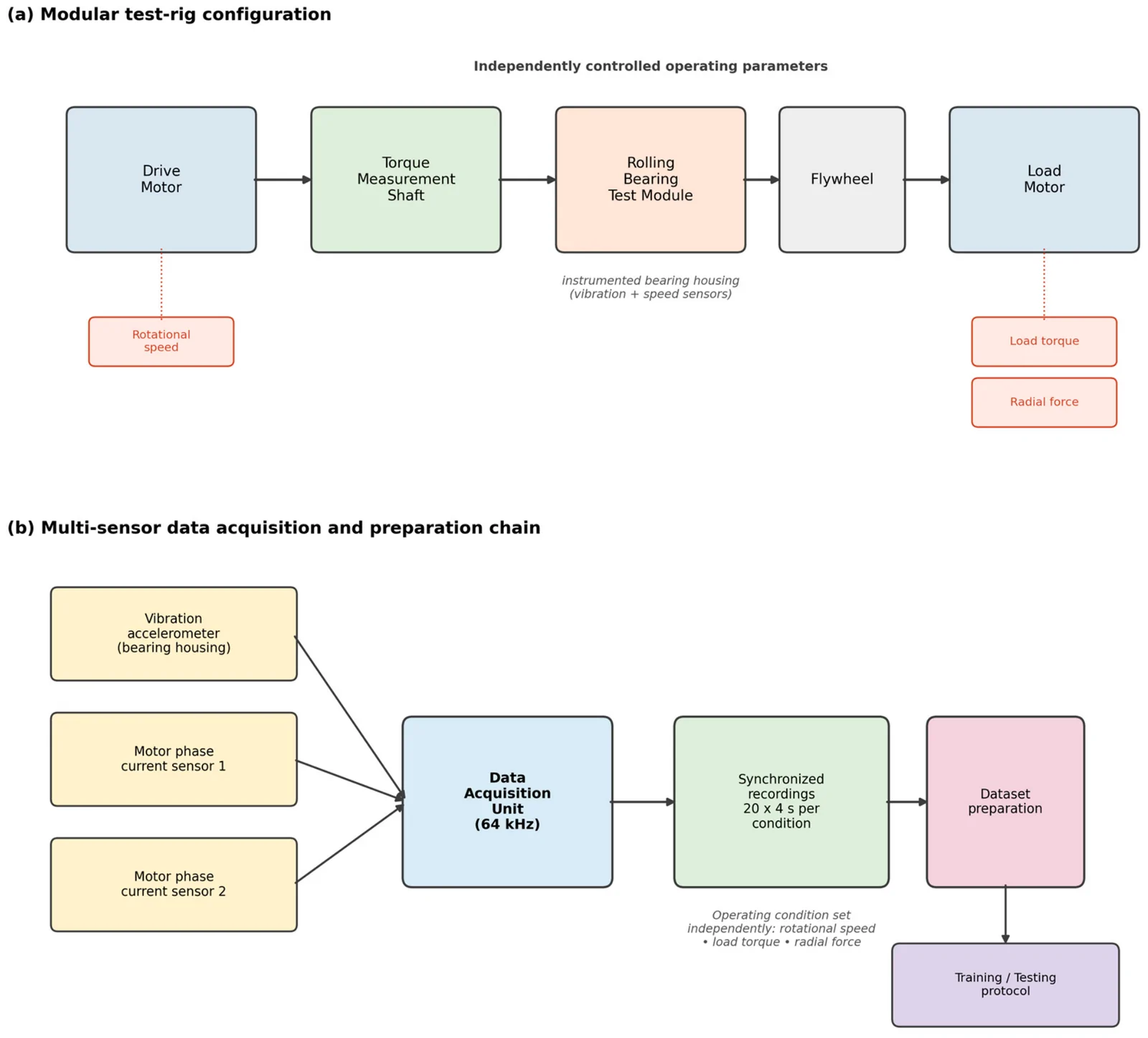
Schematic overview of (**a**) the Paderborn bearing test-rig [17] and (**b**) the multi-sensor data acquisition and preparation workflow. Solid arrows indicate the mechanical or data-flow sequence, while dashed lines indicate the association of controlled operating parameters with the corresponding test-rig components.

Data are acquired on a modular test rig comprising a drive motor, a torque measurement shaft, a rolling bearing test module, a flywheel, and a load motor, as illustrated in Figure 5.

Signals are recorded at a sampling frequency of 64 kHz over a duration of 4 s per recording, producing 256,000 samples per signal per file and 20 files per bearing per operating condition. Three signal types are used in this work: the vibration signal measured at the bearing housing (vibration_1) and the two motor phase current signals (phase_current_1 and phase_current_2). The joint use of these three sensors enriches the health state representation of the bearing; the vibration signal captures the impulsive mechanical impacts characteristic of bearing faults, while the motor currents reflect the electromechanical disturbances induced by those same faults, providing a complementary and partially redundant view of the system state.

Four health state classes are considered, representing the fault conditions most commonly encountered in industrial operation. The Healthy class corresponds to bearing K001 in its nominal operating state, serving as the reference for normal behavior. The IR class designates an inner race fault (bearing KI01), artificially induced by electro-discharge machining. The OR class designates an outer race fault (bearing KA01), also artificially induced. Finally, the ORIR class (bearing KB27) represents a combined fault simultaneously affecting both the inner and outer races.

Experiments are conducted under four operating conditions, each defined by a unique combination of rotational speed, load torque, and radial force. Condition N09_M07_F10 operates at 900 rpm with a load torque of 0.7 Nm and a radial force of 1000 N. Condition N15_M07_F10 operates at 1500 rpm under the same torque and radial force, representing a speed regime change typical of variable-speed industrial drives. Condition N15_M01_F10 maintains the speed at 1500 rpm and the radial force at 1000 N while reducing the load torque to 0.1 Nm, simulating load variations characteristic of equipment operating under fluctuating production demands. Condition N15_M07_F04 operates at 1500 rpm and 0.7 Nm with a reduced radial force of 400 N, reflecting scenarios where the mechanical load applied to the bearing changes due to shaft misalignment or varying process requirements. This set of four conditions enables a controlled evaluation of the framework’s robustness by varying one physical parameter at a time while holding the others fixed. The file distribution across classes and conditions is summarized in Table 2.

For reproducibility, Table 3 reports the manufacturer-specific geometry of the 6203 deep-groove ball bearing used in the Paderborn KAT test rig, as documented in the original benchmark description [17]. These parameters, together with the shaft rotational speed reported for each operating condition (Table 2), are sufficient to compute the characteristic fault-order values (Equations (2) and (3)) used throughout this work. Together with the operating conditions reported in Table 3 and the signal-processing and hyperparameter values described in the Data Preparation and Implementation Details sections (window length, stride, and confidence threshold, with the corresponding rationale), these data provide all information required to reproduce the characteristic fault-order calculations and the experimental protocol used in this study. All test bearings share the same nominal geometry, as bearings from the different manufacturers used in the benchmark (FAG, MTK, IBU/IBB) differ in characteristic kinematic frequency by no more than 1–2% [17].

### 4.2. Data Preparation and Processing

The Paderborn KAT dataset provides 20 independent 4 s recordings per bearing condition, sampled at 64 kHz. The train/test assignment is performed at the level of these entire recordings, prior to any window extraction: entire recordings are assigned either to the training condition or to the test condition, so that no window extracted downstream can originate from a recording split across both sets. Each recording is then segmented into non-overlapping groups of 40 consecutive sliding windows (512 points, 50% overlap), yielding 800 windows per class per operating condition. This file-level sampling strategy limits temporal autocorrelation between training samples while ensuring sufficient coverage of fault cycle patterns (40 windows capture approximately 40 BPFI impulse occurrences at 1500 rpm). Because window extraction is performed only after this recording-level assignment, no sliding window originating from the same physical recording can appear in both the training and the test set. This eliminates, by construction, the temporal window leakage identified by [35] as a source of inflated performance estimates in the bearing fault diagnosis literature. Consequently, the training and testing sets are composed of disjoint physical recordings acquired during independent acquisition sessions, ensuring that no temporal continuity exists across the train/test boundary. Normalization is performed using zero-mean unit-variance scaling with statistics computed exclusively on the training condition, preventing any distributional leakage from the test set. The cross-condition evaluation protocol assigns all data from condition A to training and all data from condition B to testing, with zero target-domain samples in the training set. This reflects a realistic deployment scenario and follows established protocols in the bearing fault diagnosis literature.

### 4.3. Experimental Protocol

#### 4.3.1. Evaluation Under Same Operating Distribution (Case 1)

A preliminary evaluation under identical training and testing conditions was conducted to assess the contribution of the different sensing modalities and to justify the sensor configuration adopted in the subsequent experiments. The selected operating condition, N15_M07_F10, corresponds to a nominal rotational speed, an intermediate load torque, and a high radial force, representing a typical operating regime of the Paderborn KAT test bench.

To ensure a rigorous evaluation protocol and prevent information leakage between samples originating from the same acquisition, the dataset was split at the file level, with 80% of the files used for training and the remaining 20% reserved for testing. Three sensor configurations were systematically investigated using the same MSCNN architecture with two convolution kernel scales (k = {3, 5}). Maintaining the same network architecture for all experiments ensures that differences in diagnostic performance can be attributed exclusively to the sensing modalities rather than to architectural variations.

Three configurations were considered. The first configuration, denoted as VIB, relies exclusively on the vibration signal (vibration_1). The second configuration, referred to as CUR, uses the two motor phase current signals (phase_current_1 and phase_current_2). Finally, the VIB + CUR configuration combines vibration and motor current measurements to construct a multimodal representation of the bearing condition.

#### 4.3.2. Evaluation Under Different Operating Conditions (Case 2)

To assess the robustness of the proposed framework under realistic deployment conditions, cross-condition experiments were conducted by training the models on a source operating condition and evaluating them on a different target condition. No samples from the target condition were used during training, thus reproducing a challenging scenario in which the diagnostic system is required to generalize to previously unseen operating regimes.

Unlike conventional studies that mainly investigate a single transfer direction, the present work systematically considers bidirectional operating-condition shifts. Three physical parameters were investigated independently, namely rotational speed, load torque, and radial force. For each parameter, both forward and reverse transitions were evaluated in order to account for possible directional effects.

The six investigated scenarios are summarized in Table 4.

For each scenario, one operating condition was exclusively used for training, whereas the corresponding target condition was reserved for testing. This protocol ensures the absence of target-domain information during the learning phase and provides a rigorous evaluation of the framework under varying operating conditions.

### 4.4. Implementation Details

The same implementation settings were maintained throughout all experiments to ensure a fair comparison between the investigated sensor configurations and operating-condition shifts. The principal hyperparameters used in this study are summarized in Table 5.

These hyperparameter values were selected based on established practice and physical considerations rather than exhaustive tuning. The window length (512 samples, 8 ms at 64 kHz) was chosen to be consistent with the fault-cycle coverage discussed in Section 3 (approximately one BPFI impulse per window at 1500 rpm), balancing temporal resolution against a sufficient number of training windows per file. The selected stride (256 samples, corresponding to 50% window overlap) represents a compromise between sample diversity and redundancy: it increases the number of extracted windows per file relative to non-overlapping segmentation, while still limiting the extent to which the same BPFI impulse content is duplicated across consecutive windows. The confidence threshold (T = 0.8) follows the selective prediction (reject-option) paradigm [18], in which a global confidence threshold is a standard, simple mechanism for deciding whether to accept or escalate a prediction; because the proposed ECR indicator is used comparatively across operating conditions rather than as an absolute reliability guarantee, this reduces the dependence of the conclusions on the exact numerical value of the selected confidence threshold.

During training, early stopping and learning-rate scheduling were employed to improve convergence and prevent overfitting. The same optimization strategy and network configuration were maintained throughout all experiments to ensure that the observed variations in performance originated from the investigated sensing configurations and operating-condition shifts rather than from differences in training settings.

## 5. Results and Discussion

This section reports the cross-domain diagnostic performance of the data-driven (DD-MSCNN) and physics-aware (PA-MSCNN) models across the six operating-condition shifts, and interprets the results from a resilience and deployability perspective using the indicators introduced in Section 3.6.

### 5.1. Multi-Sensor Contribution Analysis

The proposed framework relies on the joint use of vibration and motor current measurements. Before evaluating the robustness of the framework under operating-condition shifts, it is important to assess the individual contribution of each sensing modality and verify whether their combination provides a tangible diagnostic benefit.

To this end, an ablation study was conducted under identical operating conditions for training and testing. The objective was not to evaluate cross-condition robustness, but rather to isolate the intrinsic diagnostic information carried by each sensing modality. The N15_M07_F10 operating condition was selected as a representative reference regime, corresponding to nominal rotational speed, intermediate torque, and high radial load. To prevent data leakage, the train–test split was performed at the file level, with 80% of the recordings used for training and the remaining 20% reserved for testing.

Three sensing configurations were evaluated using the same MSCNN architecture and identical training parameters. The first configuration used only the vibration signal (VIB), the second used only the two motor current signals (CUR), and the third combined vibration and current measurements (VIB + CUR). By maintaining an identical architecture across all experiments, any performance variation can be directly attributed to the sensing modality rather than to differences in model complexity.

Table 6 summarizes the diagnostic performance obtained with the three sensing configurations. The results clearly indicate that no single sensing modality is optimal for all fault categories. While vibration measurements provide the highest performance for outer-race and combined defects, motor current signals exhibit greater sensitivity to inner-race defects. The best overall performance is achieved when both sensing modalities are combined, suggesting that vibration and current measurements capture complementary aspects of the fault generation mechanism.

The results demonstrate that the multimodal configuration achieves the highest overall diagnostic performance. Compared with the vibration-only configuration, the fusion of vibration and motor current signals increases the overall accuracy from 97.02% to 98.12% while improving the recall of inner-race defects from 92.50% to 95.00%. Although the gain in overall accuracy remains relatively modest, it confirms that motor current measurements contribute complementary diagnostic information rather than redundant information.

A more detailed analysis reveals that each sensing modality exhibits distinct diagnostic strengths. Vibration measurements remain the most effective source of information for outer-race and combined defects, achieving perfect recall for both OR and ORIR classes. In contrast, the current-only configuration experiences a substantial reduction in OR and ORIR detection performance, indicating that these defects generate mechanical signatures that are more directly observable through vibration measurements than through stator current analysis.

Interestingly, the opposite trend is observed for inner-race defects. The current-only configuration achieves the highest IR recall (95.83%), slightly outperforming the vibration-only configuration (92.50%). This observation is physically consistent with the electromechanical coupling mechanism of rotating machinery. Inner-race defects generate periodic torque fluctuations at the characteristic BPFI frequency, which modulate the motor electromagnetic behavior and become observable in the stator current signals. Consequently, motor current measurements capture information that is only partially represented in vibration data.

The superior performance of the multimodal configuration therefore results from the complementary nature of the two sensing modalities. Vibration measurements provide a direct observation of defect-induced mechanical impacts, whereas motor currents capture the electromechanical consequences of the same degradation phenomena. Their fusion combines these two perspectives and produces a more complete representation of the bearing health state than either sensing modality alone.

Beyond diagnostic accuracy, the integration of motor current measurements also supports the broader objectives of sustainable and energy-aware maintenance. Since stator currents are directly related to electrical power consumption, they provide an additional source of information that can potentially be exploited to assess the energetic impact of bearing degradation. This capability establishes a natural link between fault diagnosis, energy monitoring, and the Industry 5.0 perspective pursued in this work.

### 5.2. Overall Performance Under Bidirectional Operating Shifts

Before analyzing the contribution of the different feature representations and the impact of shift directionality, it is first necessary to evaluate the overall behavior of the proposed diagnostic framework under bidirectional operating-condition shifts. In addition to detailed fault classification, the framework also incorporates an early-warning module intended to provide complementary information regarding the transition from healthy to faulty conditions. Therefore, the assessment of the framework should not only consider the discrimination between specific fault categories but also its capability to reliably identify abnormal behavior and maintain diagnostic performance when training and deployment conditions differ.

The first stage is dedicated to binary fault detection and aims to distinguish healthy operating states from faulty ones. Table 7 summarizes the obtained results for the six investigated bidirectional operating-condition shifts. Overall, the detector maintained a high level of performance, with classification accuracies ranging from 81.14% to 99.44%. More importantly, all scenarios achieved a Healthy Recall of 100%, indicating that no healthy sample was incorrectly identified as faulty. This result suggests that the detector successfully differentiates operating-condition variations from genuine degradation-related behavior. Although the Fault Recall exhibits larger variations depending on the investigated shift, remaining between 74.88% and 99.25%, the majority of faulty samples were consistently identified and forwarded to the subsequent diagnostic stage.

Once abnormal behavior has been detected, Stage 2 performs detailed fault classification under cross-condition deployment. Table 8 reports the classification accuracy obtained by the data-driven branch (DD-MSCNN) and the physics-aware branch (PA-MSCNN) for the six bidirectional operating-condition shifts.

Several observations emerge from these results. First, the fault-detection stage appears substantially more stable than the fault-classification stage. While Stage 1 maintained accuracies above 90% for five of the six investigated scenarios and never dropped below 81.14%, Stage 2 exhibited considerably larger performance variations depending on the operating-condition shift. This observation suggests that detecting the presence of abnormal behavior remains generally more robust than identifying the precise fault category when operating conditions change.

Second, neither representation consistently outperforms the other. The relative performance of DD-MSCNN and PA-MSCNN varies according to both the type and the direction of the investigated shift. Data-driven features achieved the highest accuracy in both speed-related scenarios, whereas physics-aware features consistently provided the best performance under radial-force variations and in the most challenging torque-related conditions.

Finally, the results clearly indicate that operating-condition shifts are not equivalent. Radial-force variations constitute the least disruptive deployment scenario, with both branches achieving accuracies above 93%. In contrast, the Torque-Up transition (M01 → M07) represents the most challenging case, reducing the classification accuracy to 45.27% for DD-MSCNN and 69.14% for PA-MSCNN. Such differences suggest that the impact of an operating-condition change depends not only on its magnitude but also on its physical nature. This observation motivates the more detailed resilience-oriented analyses presented in the following sections.

To provide a more comprehensive evaluation than classification accuracy alone, and to explicitly account for class-balanced performance across the three Stage-2 fault categories (IR, OR, and ORIR), Table 9 reports the macro-averaged precision, recall, and F1-score for both branches across the six bidirectional operating-condition shifts. Macro-averaging weights each class equally regardless of its number of samples, making it a more class-imbalance-sensitive complement to the accuracy values reported in Table 8.

Macro-averaged metrics were computed on the Stage-2 fault-only evaluation set (IR, OR, and ORIR), consistent with the Stage-2 classification protocol described in the manuscript.

The macro-averaged metrics generally confirm the accuracy-based trends while providing additional insight into the balance of class-wise performance. Under Torque-Up, DD-MSCNN achieves a macro-F1 of 0.31, confirming a severe and uneven degradation across fault classes. PA-MSCNN improves the macro-F1 to 0.54, consistent with the positive contribution of the physics-aware representation observed for this scenario. Nevertheless, the remaining class-wise degradation indicates that the achieved performance is still insufficient for autonomous deployment, thereby supporting the Class D recommendation. Conversely, under Torque-Down and the radial-force shifts, the macro-averaged metrics remain high and closely aligned across precision, recall, and F1-score, confirming that the strong performance is not driven by a single fault class. These complementary results reinforce the scenario-dependent contribution of physical descriptors rather than suggesting the universal superiority of either branch.

The results reported in Table 8 and Table 9 correspond to single-run evaluations under fixed random seeds. To assess the statistical robustness of these results, we conducted a multi-seed replication (three seeds: 42, 52, 62) on one representative scenario from each deployability class Force-Up (Class A), Speed-Up (Class B), and Torque-Up (Class D), spanning the range of task difficulty observed across the six investigated shifts, rather than repeating the full protocol on all six scenarios given the associated computational cost. For each seed, both the train/validation partition and the model initialization were varied jointly, so that the reported variability reflects the combined sensitivity of the Stage-2 classifiers to random weight initialization and stochastic optimization during neural-network training (through the Adam optimizer and data shuffling), rather than only the effect of random model initialization. Accordingly, Table 10 reports the results as mean ± standard deviation, providing an estimate of the reproducibility of the reported classification performance across independent training runs.

The results show a clear gradation in run-to-run variability across deployability classes. Under Force-Up (Class A), both branches are highly stable (SD ≤ 0.004), consistent with autonomous deployment. Under Speed-Up (Class B), DD-MSCNN remains comparatively stable (SD = 0.051) while PA-MSCNN exhibits substantially larger variability (SD = 0.228 in accuracy), with individual seeds ranging from below to above the data-driven branch, indicating that the sign of the physics contribution itself is seed-dependent for this shift. Under Torque-Up (Class D), PA-MSCNN again shows higher variability than DD-MSCNN, and achieves a higher mean accuracy overall (0.490 versus 0.353); however, this advantage is not observed at every seed, corroborating the positive average physics contribution discussed above while confirming that this benefit is not uniformly reproducible across individual runs. These findings reinforce the proposed deployability classification with an additional statistical-robustness dimension: the representative Class A scenario is stable as well as high-performing, whereas the representative Class B and Class D scenarios combine lower or more variable performance with reduced run-to-run reproducibility, particularly for the physics-aware branch. A systematic multi-seed replication across all six scenarios was not conducted due to computational cost and is identified as a direction for future work.

### 5.3. Comparative Analysis of Data-Driven and Physics-Aware Representations

**Figure 6:**
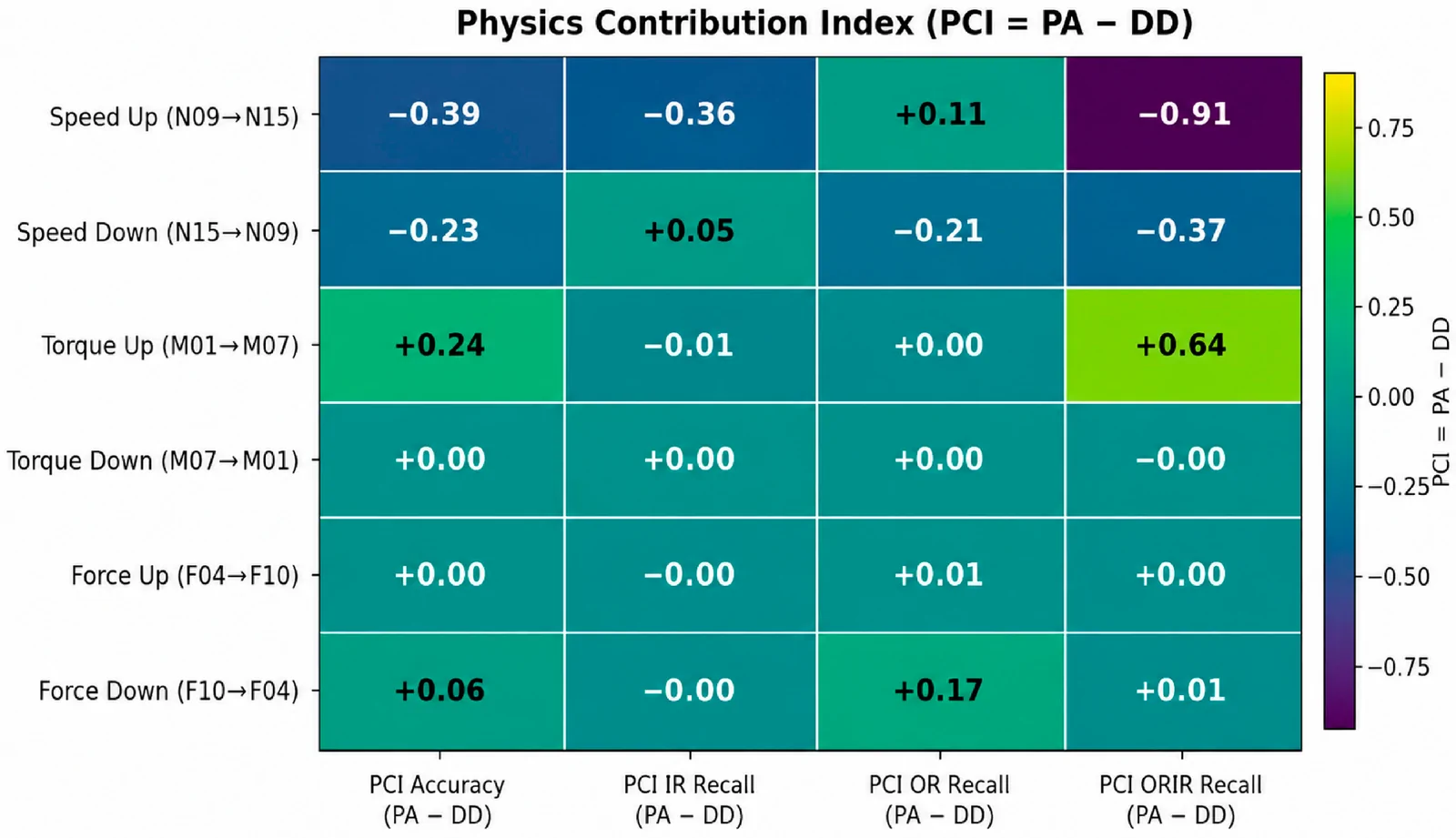
Physics contribution index heatmap.

To better understand the relative contribution of the data-driven and physics-aware representations, the Physics Contribution Index (PCI) was analyzed. The PCI heatmap illustrated in Figure 6 shows that the benefit of the physics prior is conditional on the shift rather than uniform. For radial-force shifts, all PCI values are near zero (|PCI| ≤ 0.17 on any class, ≤0.06 globally): when the domain gap is small, both models saturate, and the prior adds little. The prior becomes decisive in two opposite directions for the load-related regimes. In the torque-increase shift M01 → M07, the PA-MSCNN raises global accuracy by +0.24 and ORIR recall by +0.64 over DD, the largest positive contribution observed. Conversely, the speed-increase shift N09 → N15 shows the largest negative contribution, the PA-MSCNN losing 0.91 of ORIR recall and 0.36 of IR recall relative to DD.

This asymmetry indicates that the physics regularisation aligns with load-driven spectral signatures (torque) but is counter-productive under kinematic change (speed), where it appears to bias the model toward an incorrect class prior. Physics augmentation should therefore be treated as a targeted tool: most beneficial precisely where the data-driven model fails to transfer (torque loading), and best disabled or down-weighted under strong speed variation.

To better understand the origin of these PCI variations, the confusion matrices of DD-MSCNN and PA-MSCNN were further analyzed for each operating-condition shift. While the PCI quantifies whether physics-aware descriptors improve or degrade diagnostic performance, the confusion matrices reveal which fault classes are responsible for these changes and provide insight into the underlying decision boundaries learned by the models.

**Figure 7:**
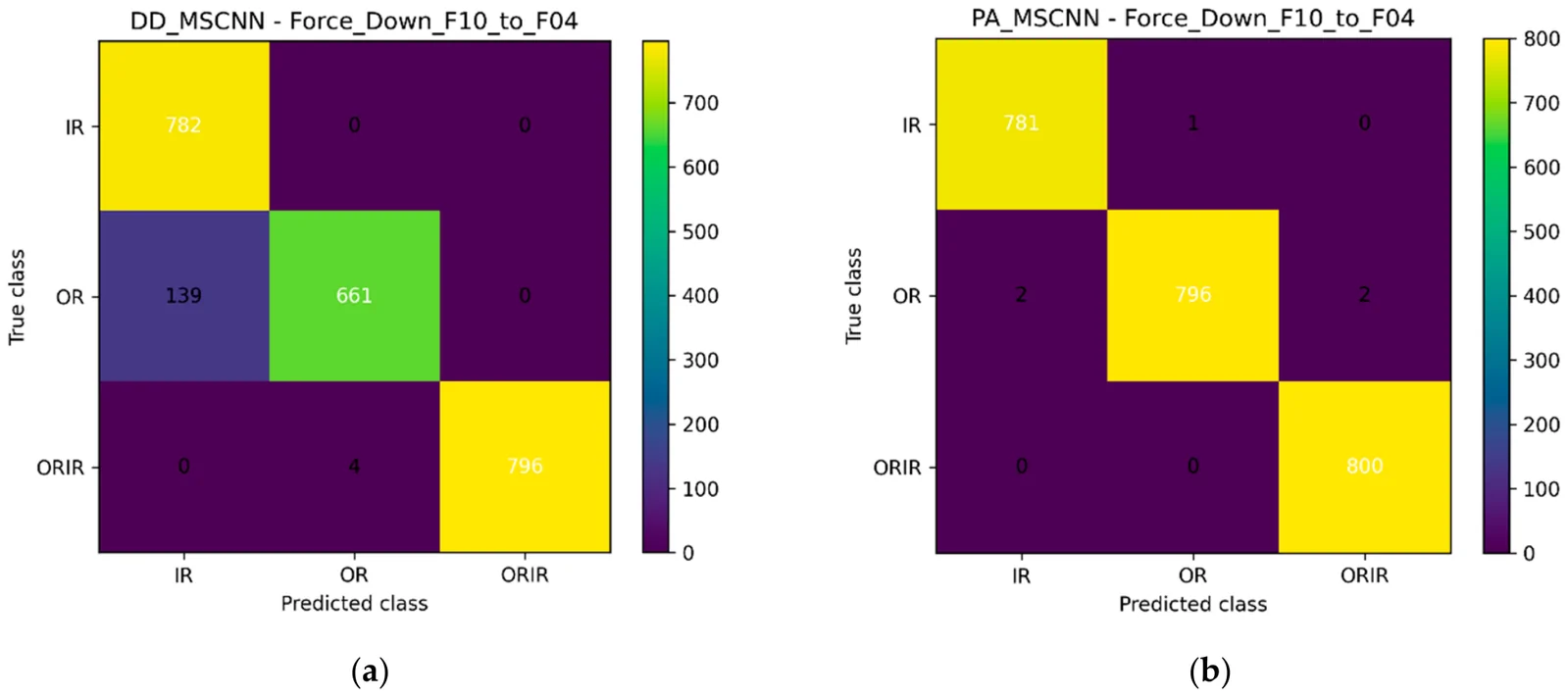

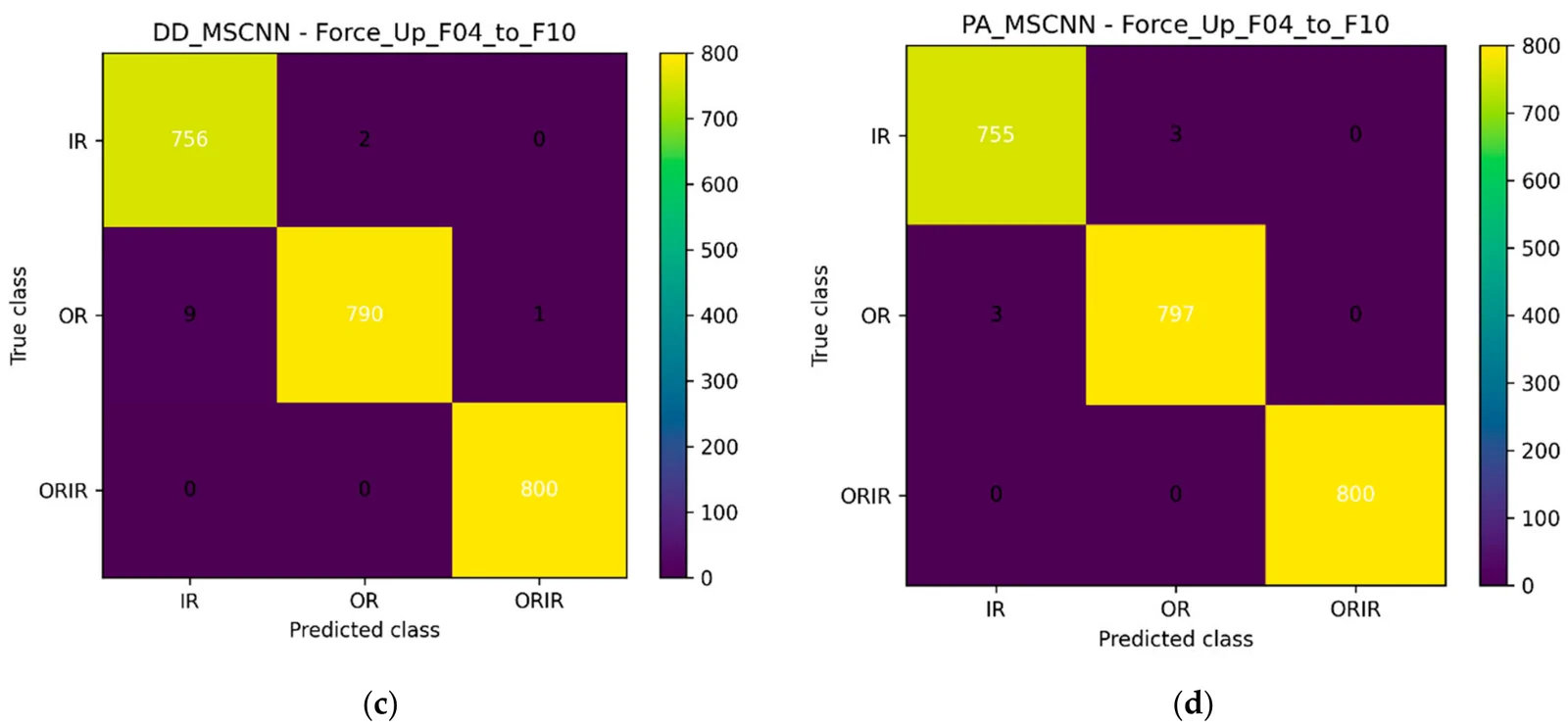
Confusion matrices of DD-MSCNN and PA-MSCNN under radial-force operating-condition shifts (F04 → F10 and F10 → F04). (**a**) DD-MSCNN–Force Down (F10 → F04). (**b**) PA-MSCNN–Force Down (F10 → F04). (**c**) DD-MSCNN–Force Up (F04 → F10). (**d**) PA-MSCNN–Force Up (F04 → F10).

For the radial-force shifts (F04 → F10 and F10 → F04), both representations exhibit nearly perfect class separation, as illustrated in Figure 7. The ORIR class remains almost perfectly identified by both models, confirming that radial-force variations have a limited impact on the discriminative structure of the feature space. The only noticeable difference appears during the Force Down scenario, where DD-MSCNN exhibits a residual confusion between OR and IR faults. This confusion is almost entirely removed by PA-MSCNN, explaining the slight positive PCI observed for this scenario. Overall, the confusion matrices confirm that radial-force variations generate only a modest domain shift and therefore do not require strong physical regularization.

**Figure 8:**
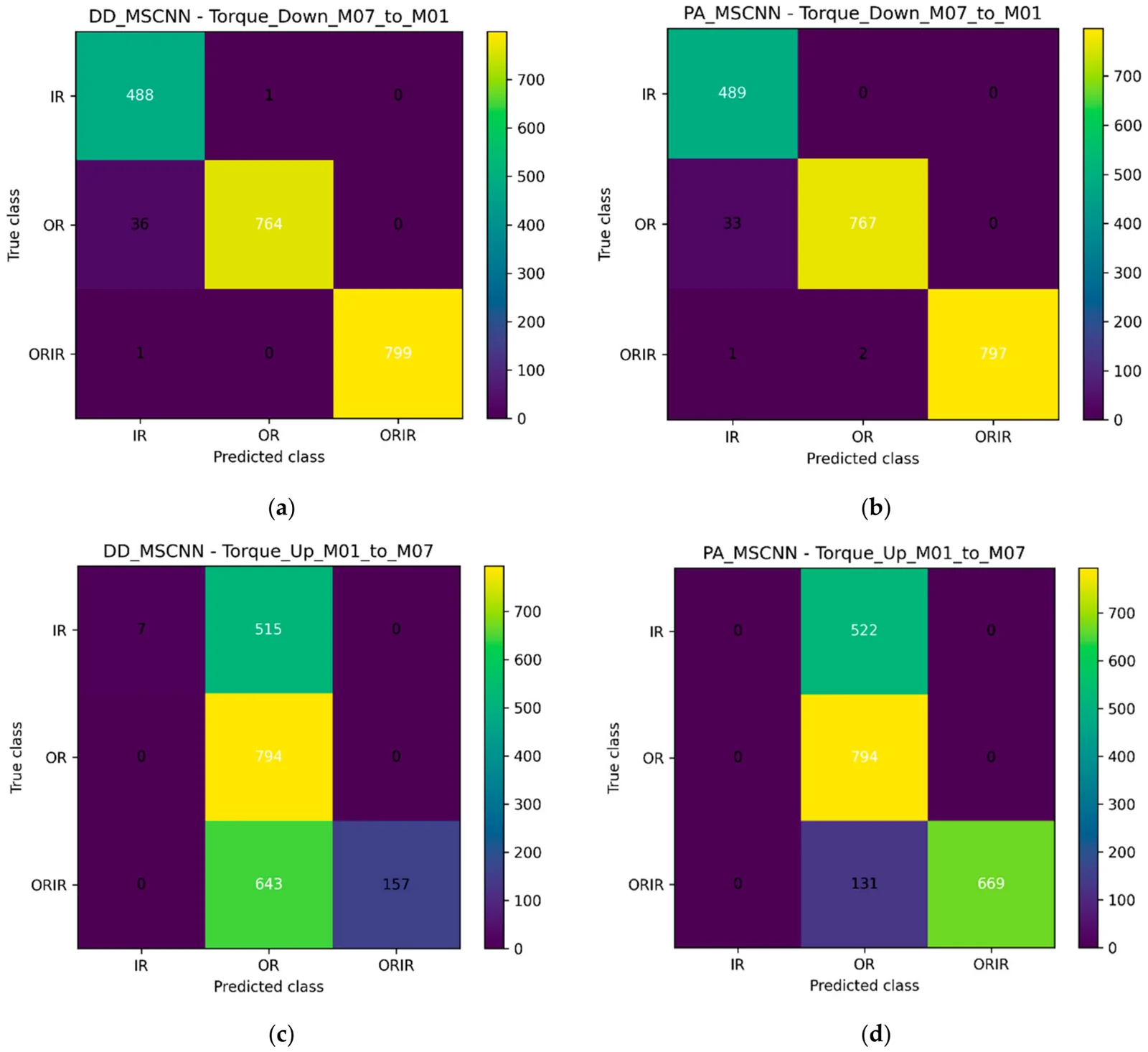
Confusion matrices of DD-MSCNN and PA-MSCNN under Torque operating-condition shifts (M01 → M07 and M07 → M01). (**a**) DD-MSCNN–Torque Down (M07 → M01). (**b**) PA-MSCNN–Torque Down (M07 → M01). (**c**) DD-MSCNN–Torque Up (M01 → M07). (**d**) PA-MSCNN–Torque Up (M01 → M07).

A markedly different behavior is observed under torque variations (Figure 8). While the Torque Down scenario (M07 → M01) remains relatively well separated for both representations, the Torque Up scenario (M01 → M07) constitutes the most challenging load-related condition. In this case, both models experience a severe degradation in the identification of inner-race defects. Most IR samples are reassigned to the OR class, indicating a substantial overlap between the corresponding feature distributions. However, the behavior of the compound ORIR fault differs significantly. DD-MSCNN correctly identifies only 157 ORIR samples, whereas PA-MSCNN successfully recovers 669 samples. This improvement directly explains the large positive PCI obtained for ORIR under Torque Up and suggests that the order-domain descriptors preserve useful fault-related information when increasing torque alters the spectral energy distribution of the signals.

Nevertheless, the persistence of severe IR-OR confusion in both representations under Torque Up suggests a physical limitation shared by data-driven and physics-aware features alike. An increase in load torque directly modifies the radial preload and the load-zone distribution experienced by both inner- and outer-race defects, which primarily alters the amplitude-domain energy of the fault-induced impacts rather than their characteristic order-domain signature. Because the physics-aware descriptors are constructed primarily from order-tracking and BPFI/BPFO-related information, they are well suited to disambiguating faults whose separability is driven by frequency-domain shifts, but order-tracking descriptors may not fully discriminate between these fault signatures under increased load. This suggests that resolving the IR-OR confusion under torque increase may benefit from complementary load-sensitive physical descriptors that explicitly characterize amplitude modulation induced by contact-force variations, in addition to order-domain information. This interpretation is consistent with the complementary role of the physics-aware branch discussed throughout the manuscript: physics-aware descriptors provide complementary rather than universally superior information, and their contribution depends on the physical nature of the operating-condition shift.

**Figure 9:**
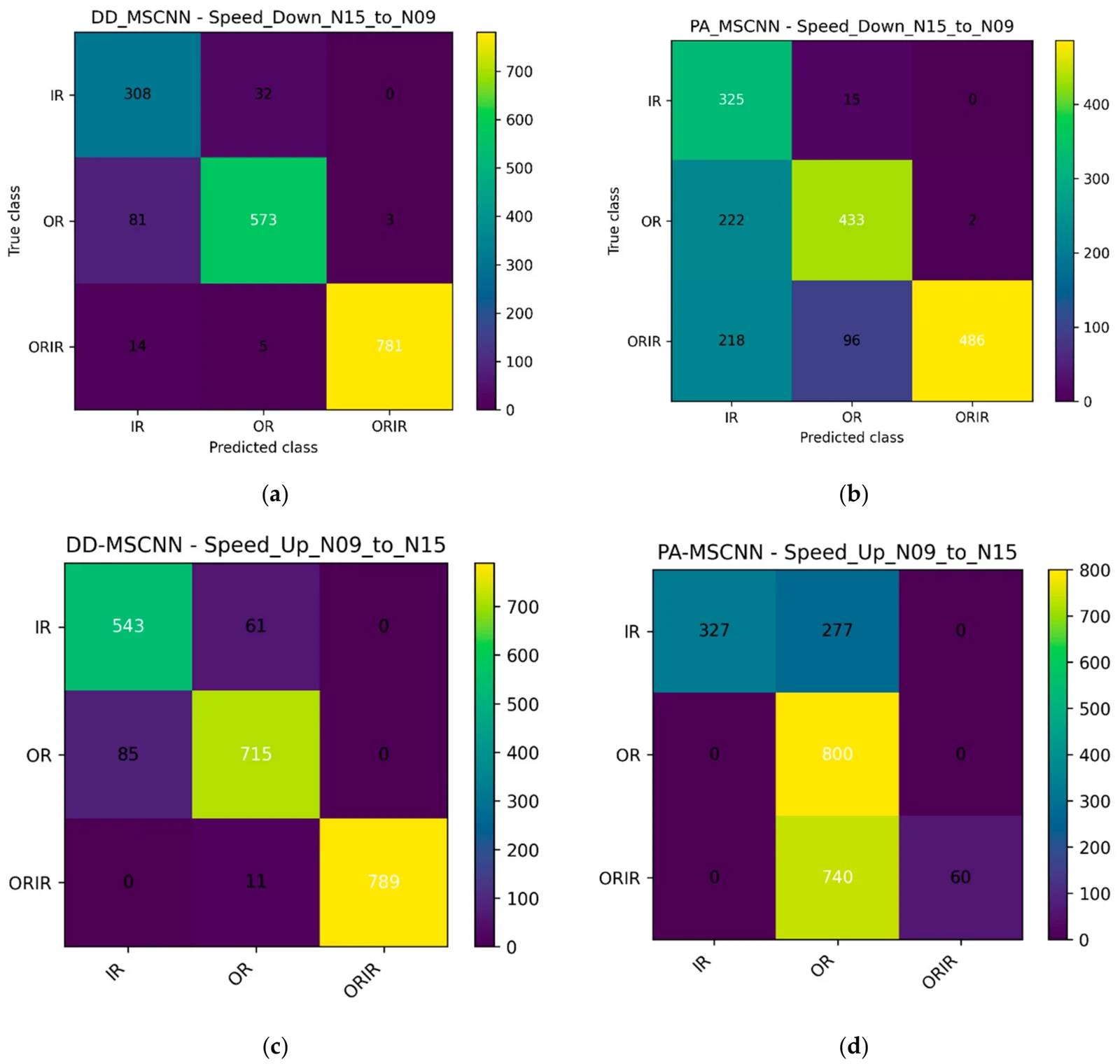
Confusion matrices of DD-MSCNN and PA-MSCNN under Speed operating-condition shifts (N09 → N15 and N15 → N09). (**a**) DD-MSCNN-Speed Down (N15 → N09). (**b**) PA-MSCNN Speed-Down (N15 → N09). (**c**) DD-MSCNN-Speed Up (N09 → N15). (**d**) PA-MSCNN-Speed Up (N09 → N15).

The most significant differences between DD-MSCNN and PA-MSCNN are observed under speed variations (Figure 9). During the Speed Down scenario, both models exhibit a moderate degradation of class separability, with most errors occurring along the IR/OR boundary. However, the Speed Up scenario reveals a much stronger divergence between the two representations. DD-MSCNN maintains a relatively balanced classification performance across the three fault categories, whereas PA-MSCNN exhibits a severe collapse of the ORIR class. More than 90% of ORIR samples are reassigned to the OR category, resulting in the largest negative PCI observed in this study. This behavior indicates that the selected order-tracking descriptors do not remain equally discriminative under large speed increases. Although order normalization compensates for frequency shifts, it appears to reduce the separability between compound and outer-race defects, ultimately degrading the performance of the physics-aware representation.

These results demonstrate that the effectiveness of physics-aware descriptors depends not only on the fault category but also on the physical mechanism responsible for the operating-condition change. Torque variations primarily affect signal amplitudes and energy distributions, for which order-based descriptors provide useful normalization. In contrast, speed variations modify the dynamic excitation mechanisms of the system and may alter the relationship between characteristic defect frequencies and the surrounding spectral content. Under these conditions, the purely data-driven representation retains greater flexibility and adapts more effectively to the modified signal structure.

### 5.4. Impact of Shift Direction on Diagnostic Robustness

Operating-condition shifts are often characterized solely by the physical nature of the operating-condition change, implicitly assuming that the transfer difficulty remains identical regardless of the direction of the transition. However, the bidirectional evaluation protocol adopted in this study reveals that this assumption is not always valid. To quantify the asymmetry between forward and reverse operating-condition transitions, the Shift Directionality Index (SDI) was introduced. In addition to overall diagnostic accuracy, class-wise recall values were considered when computing the SDI for the IR, OR, and ORIR fault categories. Recall was selected because the primary objective of the directionality analysis is to evaluate the preservation of fault detectability under opposite operating-condition transitions. In predictive maintenance applications, failing to identify an existing fault is generally more critical than generating an occasional false alarm. Consequently, recall provides a direct measure of the ability of the diagnostic system to maintain fault recognition capability when the operating condition changes. This choice is particularly relevant in the present study, where certain fault categories experience severe degradation despite relatively stable overall accuracy values.

Table 11 summarizes the SDI values obtained for the three investigated operating-condition regimes. An SDI value close to zero indicates that both transfer directions exhibit similar diagnostic behavior, whereas larger values reveal a pronounced directional effect.

The results show that the influence of shift direction strongly depends on the physical nature of the operating-condition variation. Radial-force shifts exhibit the lowest SDI values across all evaluated metrics. The maximum SDI remains below 0.01, indicating nearly identical diagnostic performance for both Force Up (F04 → F10) and Force Down (F10 → F04) scenarios. This observation confirms the robustness already highlighted in the multi-sensor contribution and overall performance analyses and suggests that radial-force variations induce relatively symmetric modifications of the fault signatures.

A different behavior is observed for speed-related shifts. Although both transfer directions remain deployable, the SDI reaches 0.135 for the global accuracy metric, indicating a moderate asymmetry between Speed Up and Speed Down transitions. Interestingly, the upward transition (N09 → N15) produces better overall performance than the reverse transition. This result suggests that increasing rotational speed does not affect the learned feature space in the same manner as decreasing speed, despite the comparable magnitude of the operating-condition change.

The strongest directional effect is observed for torque-related shifts. While the global accuracy SDI reaches 0.207, the asymmetry becomes much more pronounced when individual fault classes are considered. The inner-race defect exhibits an SDI of 0.987, representing the highest directional sensitivity observed in the entire study. This behavior originates from the severe degradation of IR classification during the Torque Up scenario (M01 → M07), whereas the reverse transition (M07 → M01) maintains almost perfect IR recognition.

The confusion matrices presented in the comparative analysis of the two representations further support this observation. Under increasing torque conditions, both DD-MSCNN and PA-MSCNN experience a near-complete collapse of the IR decision boundary, causing most IR samples to be reassigned to the OR class. In contrast, the reverse transition preserves the separability of the three fault categories. Consequently, the difficulty of the transfer process cannot be explained solely by the distance between operating points; the direction of the transition itself plays a critical role.

These findings have important practical implications for industrial deployment. A diagnostic model validated under decreasing-load conditions cannot be assumed to exhibit equivalent robustness under increasing-load conditions. Similarly, successful transfer from a high-speed regime to a low-speed regime does not guarantee comparable performance in the opposite direction. Therefore, robustness evaluation should explicitly consider bidirectional operating-condition transitions rather than relying on a single transfer direction.

Overall, the SDI analysis demonstrates that shift direction constitutes an independent source of diagnostic uncertainty. The results suggest that deployment-oriented evaluation protocols should account for both the magnitude and the direction of operating-condition changes to avoid overestimating model robustness in real industrial environments.

### 5.5. Deployability Assessment and Resilience Analysis

#### 5.5.1. Confidence and Autonomous Acceptance Analysis

Although classification accuracy provides valuable information regarding diagnostic performance, it does not fully characterize the reliability of autonomous decisions. In practical industrial applications, accurate predictions are not necessarily synonymous with trustworthy decisions, particularly when operating conditions differ from those encountered during training.

To address this limitation, the confidence-aware analysis introduced earlier is applied across all six operating-condition shifts. Samples associated with insufficient confidence or disagreement between the DD-MSCNN and PA-MSCNN branches are identified as escalation candidates. The proportion of such samples is quantified by the Escalation Candidate Rate (ECR).

Table 12 summarizes the confidence-related indicators obtained for the six operating-condition shifts, including the proportion of low-confidence predictions for each representation, the disagreement rate between both branches, and the resulting ECR values.

The results reveal that high classification accuracy does not necessarily imply high confidence. Several scenarios that achieve satisfactory diagnostic accuracy (Table 8) still present relatively high ECR values; for instance, the speed shifts exhibit ECR values close to 58% despite acceptable accuracies, indicating that a substantial proportion of predictions cannot be considered sufficiently reliable for fully autonomous acceptance. Conversely, the force shifts and the Torque-Down scenario combine low disagreement rates with low ECR values (below 17%), reflecting stronger confidence and greater resilience to operating-condition variations.

Therefore, the ECR complements conventional performance metrics by providing additional information regarding the reliability of autonomous decisions, thereby contributing to bridging the gap between laboratory evaluations and practical deployment requirements.

#### 5.5.2. Deployability Recommendations

Based on the combined analysis of classification performance, confidence indicators, physical contribution, and directional effects associated with operating-condition variations, practical deployment recommendations can be formulated for each operating scenario. The resulting deployability levels and preferred diagnostic branches are summarized in Table 13.

As shown in Table 13, force-related transitions and the Torque-Down scenario exhibit the highest level of resilience, combining high diagnostic performance with low uncertainty levels. These scenarios can therefore be considered suitable for autonomous deployment.

Speed-related transitions maintain satisfactory diagnostic capabilities but are associated with moderate uncertainty levels. Consequently, deployment remains feasible, although periodic monitoring is recommended to ensure reliable operation under varying speed conditions.

In contrast, the Torque-Up scenario exhibits substantial performance degradation together with increased uncertainty. Therefore, this scenario cannot be considered suitable for direct deployment and would require additional adaptation mechanisms or target-condition information before practical implementation.

More generally, the PCI values obtained across the six scenarios (Section 3) indicate that the contribution of physical knowledge is not uniform but depends on the physical nature of the operating-condition shift: near-neutral for radial-force shifts, most strongly positive for the torque-increase shift discussed above, where PA-MSCNN provides the largest positive contribution observed in this study and, conversely, detrimental under the speed-increase shift for the run reported in Table 8; the multi-seed analysis in Table 10 further shows that the direction of this contribution is itself seed-dependent for this shift, ranging from strongly detrimental to mildly beneficial. The residual IR-OR confusion discussed above therefore does not indicate that the physics-aware branch fails at Torque-Up; on the contrary, it is precisely where physical knowledge contributes most, while still leaving room for further improvement through complementary descriptors. This dependency illustrates the complementary, rather than uniformly superior, role of the physics-aware branch discussed throughout the manuscript.

#### 5.5.3. Complementary Evaluation of the Deployability Classes Using Supervised Transfer Learning

To further examine the practical meaning of the deployability classes reported in Table 13, three representative supervised transfer-learning configurations: frozen backbone, partial fine-tuning, and full fine-tuning, were evaluated using limited labeled samples from each target operating condition. Two of these configurations, frozen backbone and partial fine-tuning, follow the taxonomy established in recent multimodal bearing fault classification work [54]; full fine-tuning, involving no frozen layers, was additionally included as a standard upper-bound baseline for comparison. Experiments were conducted at 5%, 10%, and 20% labeled target samples; Table 14 reports the 5% setting, selected as the most restrictive labeled-data budget and therefore the closest in spirit to the target-free philosophy of the proposed framework. Unlike DD-MSCNN and PA-MSCNN, which are evaluated without target-domain samples or target-domain retraining, the transfer-learning configurations use labeled target data for supervised adaptation. The comparison therefore provides a complementary empirical check of whether the proposed deployability classes are consistent with the performance gain obtainable when limited target-domain supervision becomes available.

The results in Table 14 show a clear gradation in the supervised-adaptation gain. Class A scenarios exhibit negligible gains relative to the best target-free branch: −0.03 percentage points for Force-Down, +0.01 for Force-Up, and +1.04 for Torque-Down. Class B scenarios exhibit moderate and scenario-dependent gains of +3.55 percentage points for Speed-Down and +4.57 for Speed-Up; moreover, partial fine-tuning under Speed-Up remains below the target-free DD-MSCNN result and presents comparatively high variability. In contrast, the Class D Torque-Up scenario exhibits a substantially larger gain, with accuracy increasing from 69.14% for the best target-free branch to 95.08% under full fine-tuning, corresponding to a 25.94 percentage-point gain. These findings are consistent with the deployment recommendations in Table 13: adaptation provides little additional value in Class A scenarios, a moderate benefit in Class B scenarios, and a substantial benefit in the Class D scenario. Given that this analysis covers six operating-condition shifts from a single dataset, it should be interpreted as complementary empirical support rather than as universal validation of the proposed classes.

Overall, these results demonstrate that deployability should not be evaluated solely from classification accuracy. Reliable industrial deployment requires simultaneously considering prediction performance, confidence levels, physical contribution, and the directional effects associated with operating-condition changes. Such a multi-perspective analysis provides a more realistic evaluation of diagnostic resilience and contributes to the development of trustworthy Industry 5.0 maintenance systems.

#### 5.5.4. Computational Cost Analysis

To assess the computational feasibility of the proposed framework for real-time industrial deployment, the trainable parameter count and single-sample inference latency of DD-MSCNN and PA-MSCNN were measured on the same CPU platform using TensorFlow 2.12.0 (Google LLC, Mountain View, CA, USA). The corresponding computational complexity and latency results are summarized in Table 15. Latency measurements were performed with a batch size of one over 300 timed forward passes following 10 warm-up passes. The reported latency corresponds exclusively to neural-network inference and excludes the extraction of order-domain descriptors required by PA-MSCNN, which is performed during the signal preprocessing stage.

DD-MSCNN contains 13,507 trainable parameters, whereas PA-MSCNN contains 14,691, corresponding to an 8.77% increase. The mean inference latency increased only marginally from 40.033 ms to 40.089 ms (+0.056 ms, 0.14%). The 95th percentile latency (p95), which represents the latency below which 95% of inference executions are completed, remained nearly unchanged (62.376 ms versus 62.403 ms). These results indicate that the physics-aware sub-network introduces only a limited increase in model complexity and inference cost relative to the purely data-driven branch. With a sliding-window stride of 256 samples at a 64 kHz sampling rate, a new window becomes available approximately every 4 ms. On the evaluated CPU platform, the measured inference latency exceeds this interval for both branches, indicating that direct sequential processing of every overlapping window would require additional optimization. Nevertheless, this does not preclude industrial deployment. Practical implementations may reduce window overlap, process windows in batches, perform window subsampling, or leverage dedicated hardware acceleration (e.g., GPUs or edge AI accelerators) to match the incoming data rate.

## 6. Conclusions

This work proposed a resilience-oriented framework for fault diagnosis under varying operating conditions. Unlike conventional approaches that mainly focus on maximizing classification accuracy under fixed conditions, the proposed methodology explicitly investigates how operating-condition changes influence diagnostic behavior and how complementary sources of information can be exploited to improve reliability.

The framework combines a Multi-Stage diagnosis strategy with two complementary representations. A data-driven branch based on multi-scale convolutional neural networks and a physics-aware branch integrating order-tracking descriptors were developed and evaluated under six bidirectional operating-condition shifts involving speed, torque, and radial force variations. In addition, a confidence-aware decision layer was introduced to analyze prediction consistency and support the assessment of diagnostic reliability.

The obtained results demonstrate that operating-condition changes are not equivalent and that their effects strongly depend on the direction of the transition. The analysis further reveals that the incorporation of physical information does not systematically guarantee superior performance. Depending on the scenario, either the data-driven or the physics-aware representation may provide the most suitable solution. These observations highlight the importance of evaluating fault diagnosis systems from a resilience perspective rather than relying solely on average classification accuracy.

To provide a deeper understanding of the framework behavior, the Physics Contribution Index (PCI) and the Shift Directionality Index (SDI) were introduced to quantify, respectively, the contribution of physical knowledge and the asymmetry between forward and reverse operating-condition shifts. Furthermore, confidence-based indicators and a deployability analysis were used to assess the practical applicability of the framework under previously unseen conditions.

Overall, the results suggest that no single representation can be considered universally optimal. Instead, diagnostic performance should be interpreted as a dynamic interaction between signal characteristics, operating conditions, and prior physical knowledge. The objective of the proposed framework is therefore not to eliminate all post-deployment interventions, but to determine, before deployment, whether autonomous operation is justified or whether additional adaptation, monitoring, or supervision should be anticipated. From an industrial perspective, the proposed framework provides not only diagnostic predictions but also additional information regarding confidence, resilience, and deployability, thereby facilitating the practical implementation of intelligent maintenance systems.

These contributions should nonetheless be considered in light of certain methodological choices. In particular, the confidence information exploited in this study is obtained directly from the softmax outputs of the diagnostic branches. Although softmax probabilities do not constitute a calibrated measure of uncertainty and may exhibit overconfidence under distribution shifts, the present work focuses on exploiting confidence information for resilience and deployability assessment rather than on developing a dedicated uncertainty estimator. The proposed framework should therefore be viewed as confidence-aware rather than uncertainty-aware in the strict Bayesian sense. More sophisticated uncertainty quantification techniques, such as Bayesian neural networks, evidential learning, or deep ensembles, could provide better-calibrated estimates and constitute a natural avenue for strengthening the confidence layer. Accordingly, the Escalation Candidate Rate (ECR) should be interpreted as a comparative decision-support indicator, used to rank operating-condition scenarios by relative reliability concern, rather than as an absolute measure of predictive uncertainty. Importantly, the overall architecture of the proposed framework is independent of the specific confidence-estimation method: should better-calibrated techniques become available, they could replace the softmax-based confidence layer without altering the general logic of the resilience and deployability assessment. It should be noted that the present work is not intended to perform a classical probabilistic reliability analysis (e.g., failure probability distributions, Weibull-based lifetime modeling, or reliability indices derived from degradation statistics). Instead, the notion of reliability adopted in this work refers to the trustworthiness of diagnostic decisions under varying operating conditions, as assessed through the proposed confidence-aware and deployability indicators (Section 3.6).

The physics-aware branch assumes the availability of rotational-speed information for order normalization. Although such information is commonly available in instrumented industrial drivetrains, extending the proposed framework to speed-sensorless environments through tacholess order-tracking or speed-estimation techniques constitutes an interesting direction for future research. The term “target-free” in this work specifically refers to the absence of target-domain measurements, labels, unlabeled recordings, or distributional information during training and deployment. The shaft rotational speed used for order normalization is a routinely available operating parameter required to express the vibration signal in the order domain, rather than target-domain measurements, labels, or distributional information, and is independent of the target-domain signal distribution. Consequently, the proposed protocol remains target-free with respect to target-domain data, although it assumes the availability of shaft rotational speed for order normalization; this distinction delimits the scope of the target-free claim and should be understood as such throughout this work.

While the physics-aware branch is here instantiated for rolling-element bearings, its underlying principle is component-agnostic. Transferring the framework to other rotating components, such as gears, by substituting the corresponding physical descriptors (e.g., gear-mesh frequency and sidebands) constitutes a natural direction for future work.

In the present work, the order-domain descriptors are extracted exclusively from the vibration signal. Since bearing defects also modulate the motor phase currents at the same characteristic fault frequencies, an interesting extension would be to derive the physics-aware descriptors from the current signals as well. This would enable a fully multi-sensor physics-aware representation and could additionally support rotational-speed estimation in tacholess settings, thereby relaxing the speed-availability assumption discussed above.

The Paderborn KAT benchmark was selected because it provides synchronized vibration and motor-current measurements together with systematically controlled variations in rotational speed, load torque, and radial force, enabling a rigorous bidirectional evaluation of operating-condition shifts across the six scenarios considered in this work. These characteristics make it particularly suitable for the resilience-oriented analysis proposed here. We nevertheless acknowledge that validating the framework on additional public datasets would further strengthen its generalizability, and this point has been explicitly identified in the Limitations section as an important direction for future work.

Building on these considerations, future work will focus on extending the proposed framework toward adaptive and self-evolving diagnostic systems. Domain adaptation and online learning strategies will be investigated to improve robustness under severe distribution shifts, while digital twin technologies and reinforcement learning mechanisms will be explored to support dynamic decision-making. In particular, future work will investigate whether the proposed deployability indicators (PCI, SDI, and ECR) remain stable across different bearing benchmarks and, more broadly, across rotating machinery datasets involving different sensor configurations and fault-generation mechanisms. Finally, the incorporation of real human expertise and the validation of the framework on industrial data collected under real operating environments represent promising directions for further research.

## Figures and Tables

**Table 1:** Comparative summary of variable-condition fault diagnosis approaches.

Reference	Journal	Year	Protocol	Method	Target Data Required
[8]	Sensors	2022	Distribution mixing	Signal-to-image fusion (time + spectral + envelope) across mixed conditions	same distribution
[15]	Sensors	2022	Distribution mixing	Image encoding + CNN across variable speeds and loads	same distribution
[40]	IEEE Transactions on Instrumentation and Measurement	2020	Cross-condition (DG)	1D-CNN + WGAN-GP critic + pseudo-labels, adversarial speed-invariant features	Multiple labeled source conditions required
[41]	IEEE Transactions on Instrumentation and Measurement	2022	Cross-condition (DA)	Multisource domain adaptation + MMD alignment	Unlabeled target required
[46]	Entropy	2022	Cross-condition (DA)	Multi-space dynamic distribution adaptation + MK-MMD + LMMD	Unlabeled target required
[43]	Advanced Engineering Informatics	2024	Cross-condition (CL)	Time-frequency contrastive learning + pseudo-labeling on target	Unlabeled target required
[44]	Sensors	2025	Source-free DA	Source-free DA via test-time training + U-Net VAE	Unlabeled target still required
[45]	Sensors	2025	Cross-condition (DA)	Dual-stream hybrid-domain adaptation + GMM pseudo-labels (Paderborn)	Unlabeled target required

**Table 2:** File distribution per class and operating condition.

Class	Bearing ID	Fault Type	N09_M07_F10	N15_M07_F10	N15_M01_F10	N15_M07_F04
Healthy	K001	No fault	20	20	20	20
IR	KI01	Inner race fault	20	20	20	20
OR	KA01	Outer race fault	20	20	20	20
ORIR	KB27	Combined IR + OR	20	20	20	20

**Table 3:** Geometry of the 6203 deep-groove ball bearing used in the Paderborn KAT test rig [17].

Parameter	Value
Bearing designation/boundary dimensions	6203 (17 × 40 × 12 mm)
Pitch circle diameter, D	28.55 mm
Rolling element diameter, d	6.75 mm
Number of rolling elements, n	8
Contact (pressure) angle, θ	0°

**Table 4:** Bidirectional operating-condition shift scenarios investigated in this study.

Shift Category	Forward Shift	Reverse Shift
Speed	N09 → N15	N15 → N09
Torque	M01 → M07	M07 → M01
Radial Force	F04 → F10	F10 → F04

**Table 5:** Summary of implementation hyperparameters.

Parameter	Value
Sampling frequency	64 kHz
Window length	512 samples
Stride	256 samples
Maximum windows per file	40
Sequence length (Stage 1)	4
Convolution kernel sizes	{3, 5}
Batch size	32 (Stage 1), 8 (Stage 2)
Optimizer	Adam
Maximum epochs	40 (Stage 1), 60 (Stage 2)
Early stopping patience	10
Reduce LR On Plateau patience	4
Dropout rate	0.30 (Stage 1), 0.25 (Stage 2)
Confidence threshold (T)	0.8

**Table 6:** Diagnostic performance obtained using different sensing configurations.

Configuration	Accuracy (%)	IR Recall (%)	OR Recall (%)	ORIR Recall (%)
VIB	97.02	92.50	100.00	100.00
CUR	89.95	95.83	66.25	78.33
VIB and CUR	98.12	95.00	100.00	100.00

**Table 7:** Stage 1 fault detection performance under bidirectional operating-condition shifts.

Shift Category	Scenario	Accuracy (%)	Healthy Recall (%)	Fault Recall (%)
Speed	N09 → N15 (Speed Up)	93.87	100.00	91.83
Speed	N15 → N09 (Speed Down)	81.14	100.00	74.88
Torque	M01 → M07 (Torque Up)	91.12	100.00	88.17
Torque	M07 → M01 (Torque Down)	90.27	100.00	87.04
Radial Force	F04 → F10 (Force Up)	98.69	100.00	98.25
Radial Force	F10 → F04 (Force Down)	99.44	100.00	99.25

**Table 8:** Classification accuracy of DD-MSCNN and PA-MSCNN across six bidirectional operating-condition shifts on the Paderborn KAT dataset.

Shift Category	Scenario	DD-MSCNN Accuracy (%)	PA-MSCNN Accuracy (%)	Best Model
Speed	N09 → N15 (Speed Up)	92.88	53.86	DD-MSCNN
Speed	N15 → N09 (Speed Down)	92.49	69.23	DD-MSCNN
Torque	M01 → M07 (Torque Up)	45.27	69.14	PA-MSCNN
Torque	M07 → M01 (Torque Down)	98.18	98.28	PA-MSCNN
Radial Force	F04 → F10 (Force Up)	99.49	99.75	PA-MSCNN
Radial Force	F10 → F04 (Force Down)	93.99	99.79	PA-MSCNN

**Table 9:** Macro-averaged precision, recall, and F1-score of DD-MSCNN and PA-MSCNN across six bidirectional operating-condition shifts.

Scenario	Model	Macro-P	Macro-R	Macro-F1
Speed-Up (N09 → N15)	DD-MSCNN	0.92	0.93	0.93
Speed-Up (N09 → N15)	PA-MSCNN	0.81	0.54	0.48
Speed-Down (N15 → N09)	DD-MSCNN	0.90	0.92	0.91
Speed-Down (N15 → N09)	PA-MSCNN	0.74	0.74	0.69
Torque-Up (M01 → M07)	DD-MSCNN	0.80	0.40	0.31
Torque-Up (M01 → M07)	PA-MSCNN	0.52	0.61	0.54
Torque-Down (M07 → M01)	DD-MSCNN	0.98	0.98	0.98
Torque-Down (M07 → M01)	PA-MSCNN	0.98	0.98	0.98
Force-Up (F04 → F10)	DD-MSCNN	0.99	0.99	0.99
Force-Up (F04 → F10)	PA-MSCNN	1.00	1.00	1.00
Force-Down (F10 → F04)	DD-MSCNN	0.95	0.94	0.94
Force-Up (F04 → F10)	PA-MSCNN	1.00	1.00	1.00

**Table 10:** Multi-seed statistical robustness analysis (mean ± standard deviation over three seeds) for one representative scenario per deployability class.

Scenario	Class	Model	Acc (Mean ± Standard Deviation (SD))	Macro-F1 (Mean ± SD)
Force-Up (F04 → F10)	A	DD-MSCNN	0.995 ± 0.004	0.995 ± 0.004
	A	PA-MSCNN	0.993 ± 0.003	0.993 ± 0.003
Speed-Up (N09 → N15)	B	DD-MSCNN	0.899 ± 0.051	0.896 ± 0.055
	B	PA-MSCNN	0.670 ± 0.228	0.601 ± 0.283
Torque-Up (M01 → M07)	D	DD-MSCNN	0.353 ± 0.019	0.205 ± 0.037
	D	PA-MSCNN	0.490 ± 0.151	0.379 ± 0.183

**Table 11:** Shift Directionality Index (SDI) for Global and Class-Level Diagnostic Performance.

Operating-Condition Shift	SDI (Overall Accuracy)	SDI (IR Recall)	SDI (OR Recall)	SDI (ORIR Recall)	Directionality Level
Radial Force (F04 ↔ F10)	0.003	0.003	0.008	0.000	Negligible
Speed (N09 ↔ N15)	0.135	0.076	0.083	0.189	Moderate
Torque (M01 ↔ M07)	0.207	0.987	0.000	0.639	Strong

**Table 12:** Confidence-related indicators and uncertainty levels under different operating-condition shifts.

Scenario	DD Low-Conf. Rate (%)	PA Low-Conf. Rate (%)	DD-PA Disagreement Rate (%)	ECR (%)
Speed-Up	9.85	17.33	47.46	57.67
Speed-Down	33.33	44.80	27.88	58.88
Torque-Up	22.73	30.29	24.72	38.23
Torque-Down	8.23	4.07	1.44	9.19
Force-Up	7.42	1.15	0.42	7.93
Force-Down	13.10	1.47	6.00	16.46

**Table 13:** Deployability Assessment and Deployment Recommendations per Operating-Condition Shift Scenario.

Operating-Condition Shift	Preferred Branch	Deployability Class	Recommendation
Force-Up (F04 → F10)	PA-MSCNN	A	Autonomous deployment
Force-Down (F10 → F04)	PA-MSCNN	A	Autonomous deployment
Torque-Down (M07 → M01)	PA-MSCNN	A	Autonomous deployment
Speed-Up (N09 → N15)	DD-MSCNN	B	Deployable with periodic monitoring
Speed-Down (N15 → N09)	DD-MSCNN	B	Deployable with periodic monitoring
Torque-Up (M01 → M07)	PA-MSCNN	D	Additional adaptation required

**Table 14:** Supervised-adaptation gain at a 5% labeled target-domain budget, cross-referenced with the proposed deployability classes.

Scenario	Class	DD (%)	PA (%)	Frozen 5% (%)	Partial 5% (%)	Full 5% (%)	Gain (pt)
Force-Down (F10 → F04)	A	93.99	99.79	99.68 ± 0.17	99.76 ± 0.26	99.76 ± 0.31	−0.03
Force-Up (F04 → F10)	A	99.49	99.75	99.76 ± 0.26	99.72 ± 0.18	99.74 ± 0.19	+0.01
Torque-Down (M07 → M01)	A	98.18	98.28	99.30 ± 0.30	99.01 ± 0.61	99.32 ± 0.25	+1.04
Speed-Down (N15 → N09)	B	92.49	69.23	96.04 ± 0.74	95.58 ± 1.49	95.99 ± 0.74	+3.55
Speed-Up (N09 → N15)	B	92.88	53.86	93.32 ± 1.28	89.63 ± 6.87	97.45 ± 0.84	+4.57
Torque-Up (M01 → M07)	D	45.27	69.14	66.04 ± 14.02	93.52 ± 2.84	95.08 ± 1.58	+25.94

**Table 15:** Computational complexity and CPU inference latency of DD-MSCNN and PA-MSCNN.

Model	Trainable Parameters	Parameter Increase	Mean Inference Latency (ms/Sample)	SD (ms)	95th Percentile Latency (ms)
DD-MSCNN	13,507	—	40.033	15.554	62.376
PA-MSCNN	14,691	+8.77%	40.089	11.945	62.403

## Data Availability

This study used the publicly available Paderborn University bearing dataset (KAt-DataCenter), accessible at https://mb.uni-paderborn.de/kat/forschung/bearing-datacenter/data-sets-and-download#c374354 accessed on 12 January 2026. No new data were created in this study. The code is available from the corresponding author upon reasonable request.
